# Construction and assessment of prediction rules for binary outcome in the presence of missing predictor data using multiple imputation and cross‐validation: Methodological approach and data‐based evaluation

**DOI:** 10.1002/bimj.201800289

**Published:** 2020-02-13

**Authors:** Bart J. A. Mertens, Erika Banzato, Liesbeth C. de Wreede

**Affiliations:** ^1^ Medical Statistics Department of Biomedical Data Sciences Leiden University Medical Center Leiden The Netherlands; ^2^ Department of Statistical Sciences University of Padova Padova Italy

**Keywords:** binary outcome, calibration, cross‐validation, multiple imputation, prediction

## Abstract

We investigate calibration and assessment of predictive rules when missing values are present in the predictors. Our paper has two key objectives. The first is to investigate how the calibration of the prediction rule can be combined with use of multiple imputation to account for missing predictor observations. The second objective is to propose such methods that can be implemented with current multiple imputation software, while allowing for unbiased predictive assessment through validation on new observations for which outcome is not yet available.

We commence with a review of the methodological foundations of multiple imputation as a model estimation approach as opposed to a purely algorithmic description. We specifically contrast application of multiple imputation for parameter (effect) estimation with predictive calibration. Based on this review, two approaches are formulated, of which the second utilizes application of the classical Rubin's rules for parameter estimation, while the first approach averages probabilities from models fitted on single imputations to directly approximate the predictive density for future observations. We present implementations using current software that allow for validation and estimation of performance measures by cross‐validation, as well as imputation of missing data in predictors on the future data where outcome is missing by definition.

To simplify, we restrict discussion to binary outcome and logistic regression throughout. Method performance is verified through application on two real data sets. Accuracy (Brier score) and variance of predicted probabilities are investigated. Results show substantial reductions in variation of calibrated probabilities when using the first approach.

## 1 INTRODUCTION

There has been much recent interest in the medical statistical, epidemiological, and even biomedical literature on calibration (training) and validation (testing) of prediction rules in the prognostic context, when multiple imputations are used to account for missing observations in the predictors. This renewed interest has been supported by (1) the emergence of easy‐to‐use packages for the generation of imputations within statistical software such as R or Stata as well as (2) the now ready availability of fast and cheap computing even with desktop configurations. This has unleashed creativity to propose and investigate various novel combinations of predictive calibration with validation approaches and imputation.

A feature of this literature is that it is predominantly algorithmic and somewhat ad hoc in nature. Wood, Royston, and White (2015), for example, focus on performance assessment and formulate various strategies. Wahl, Boulesteix, Zierer, Thorand, and van de Wiel (2016) investigate the problem of combining predictive calibration with validation and imputation but with a particular focus on bootstrapping. Generation of post hoc summaries, among which performance estimates, after multiple imputation, is discussed by Marshall, Altman, Holder, and Royston (2009). These authors also report on a literature review in recent biomedical literature on use of multiple imputation in prognostic studies. Vergouwe, Royston, Moons, and Altman (2010) discuss case studies on practical development of prognostic models with imputation, also addressing model selection. Miles (2015) contrasts prediction‐averaging versus application of Rubin's rules to combine model parameter estimates for predictive calibration with imputations.

In line with the predominantly algorithmic nature of these presentations, novel methods are developed as adaptations of—or combinations with—the multiple imputation algorithm. Indeed, multiple imputation itself tends to be presented as an algorithmic device, although it has a clear methodological foundation as an approximation of the joint density of effect estimates near the mode. Multiple imputation is *model* estimation. By reestablishing focus on multiple imputation as a model approximation and thus estimation approach, it may become more easy to identify suitable approaches for method validation by formulating validation as model assessment and similarly for the definition of the predictive approach itself.

In so far as consensus exists within the literature on the fundamental challenges posed by multiple imputation in prognostic calibration, it is that while imputation must take into account observed outcomes, unbiased validation by definition requires outcomes to be omitted when calibrating models; Wood et al. (2015, p. 615); Wahl et al. (2016, p. 2). A less recognized issue is that multiple imputation in the predictive context requires calibration of a distinct predictive density than is currently implemented in existing imputation software, which is focused on effect estimation instead.

To elucidate these issues, our paper commences with a methodological review of multiple imputation with special emphasis on the distinction between prediction and effect estimation in the imputation context (Section 2). Based on this discussion, we propose two basic approaches for the calibration of prognostic rules with multiple imputations that can be implemented with existing imputation software and allow for validation using a set‐aside validation set excluding outcome data (Section 3). The second of these is based on classical Rubin's rule estimation, while the first utilizes an approximation to the predictive density of future outcome. This discussion may be viewed as a formalization of the methods suggested by Miles (2015). In contrast to the above discussed existing literature, which predominantly focuses on simulation (Vergouwe's paper being a notable exception), we subsequently present a data‐based application using two real data sets from our own consultative experience, which have motivated our interest in this research. To compare methods, we study data‐based summary statistics, specifically predictive accuracy and variance based on application of methods to the data (Sections 4 and 5). We finish with a review of main results and key conclusions, and formulate recommendations.

## 2 METHODOLOGY

### 2.1 Parameter estimation and Rubin's rules

To formalize our discussion, we assume a substantive prediction model specified by a density f(Y∣X,β), which describes the variation in a vector $Y={\left(Y_{1},\ldots ,Y_{n}\right)}^{T}$ of outcomes of interest on a sample of *n* patients, conditional on a corresponding general matrix of predictors X and depending on an unknown vector of regression parameters β. The latter will need to be estimated from sampled data, prior to subsequent use of the model for future patients. We only consider scenarios with missing data in the predictors in this paper, such that $X=(X_{m},X_{o})$, which separates into missing $X_{m}$ and observed $X_{o}$ components and with *Y* fully observed. If our primary interest were to reside in the regression parameter vector β, then we would seek to estimate the conditional (so‐called posterior) density

(1) $$\begin{array}{cc} p(\beta |X_{o},Y) & =\int p\left(\beta ,X_{m}|X_{o},Y\right)dX_{m}\\ & =\int p\left(\beta |X_{m},X_{o},Y\right)p\left(X_{m}|X_{o},Y\right)dX_{m}, \end{array}$$

which is obtained as the marginalized joint posterior density on the two unknown components β and $X_{m}$, marginalized across the nuisance unobserved covariate values in $X_{m}$. The last equality reveals this may also be thought of as the probability density for the target parameter of interest β, conditional on the unknown quantities $X_{m}$, averaged across the uncertainty in $X_{m}$ (but always conditional on the actually observed data). This latter equality explains the workings of classical multiple imputation, as it generates imputed data from the conditional density $p(X_{m}|X_{o},Y)$, for each of which simulations may be generated from the corresponding densities $p(\beta |X_{m},X_{o},Y)$, to approximate the moments of $p(\beta |X_{o},Y)$ in a sampling‐based manner. The Rubin's rules‐based approach represents a practical compromise to achieve this averaging, by first sampling imputations ${\hat{X}}_{m,k}$ drawn from $p(X_{m}|X_{o},Y)$ and with k=1,…,K for a total number of *K* imputations. Subsequently, we estimate the modes ${\hat{\beta }}_{k}$ of the conditional densities $p(\beta |{\hat{X}}_{m,k},X_{o},Y)$ evaluated at the completed data sets $\left({\hat{X}}_{m,k},X_{o},Y\right)$ and for all *k*. Large‐sample results from classical frequentist theory are then used to approximate the conditional density $p(\beta |X_{o},Y)$ at the mode, and these results give rise to the so‐called Rubin's rule estimate of the expectation as

(2) $${\hat{\beta }}_{MI}=\frac{1}{K}\sum_{k=1}^{K}{\hat{\beta }}_{k}.$$

Readers can consult Carpenter and Kenward (2013, pp. 46–48), Carlin (2015) or Gelman, Carlin, Stern, and Rubin (2004, pp. 519–523), among many other sources for further results and details. Our notations in the above and following are also loosely based on those of Carpenter and Kenward (2013, e.g., pp. 44–48).

### 2.2 Prediction, the predictive density, and imputation

In the predictive scenario, the averaging described in Equation (1) no longer suffices and should be expanded to average across the regression coefficients, in order to account for *both* the missing values $X_{m}$, and the uncertainty in β. Let $\tilde{Y}$ be a future univariate outcome, which we want to predict from covariates $\tilde{X}$. As before, we have available a previous sample of data from the sample population, with outcomes *Y* and covariates X, which we will refer to as the calibration data. To simplify the discussion and notations, we will in the first instance assume that there are no further missing values in the predictor data $\tilde{X}$, such that we can write $p(\tilde{Y}|X_{o},Y)$ for the predictive density of future outcomes, which denotes the conditional dependence on the past observed calibration data $X_{o},Y$, while ignoring the obvious dependence on $\tilde{X}$ for the time being.

In analogy to the previous section, to predict future outcomes $\tilde{Y}$, we must calibrate the *predictive density*

(3) $$\begin{array}{cc} p(\tilde{Y}|X_{o},Y) & =\int f\left(\tilde{Y},\beta ,X_{m}|X_{o},Y\right)d\beta dX_{m}\\ & =\int f\left(\tilde{Y}|\beta ,X_{m},X_{o},Y\right)p\left(\beta ,X_{m}|X_{o},Y\right)d\beta dX_{m}; \end{array}$$

see Seber (1984, p. 292) and Lessafre (2012, p. 53). The last line shows that the integration can now be achieved by averaging across both imputations ${\hat{X}}_{m,k}$ and simulations ${\hat{\beta }}_{k}$ from the density $p(\beta ,X_{m}|X_{o},Y)$, while conditioning on the observed calibration data $X_{o},Y$. In analogy to Equation (1), this implies we may calculate the expectations ${\hat{P}}_{k}=E\left(\tilde{Y}|\hat{\beta_{k}},{\hat{X}}_{m,k},X_{o},Y\right)$ for each pair of imputed values $\hat{\beta_{k}},{\hat{X}}_{m,k}$, from the conditional density $p(\beta ,X_{m}|X_{o},Y)$. The set of predictions ${\hat{P}}_{k}$, k=1,…,K, may then be summarized using the mean in analogy to Rubin's rules, medians, or some other suitable summary measure to get the final prediction estimate $\hat{P}$. For example, using Rubin's rules to summarize the set of predictions ${\hat{P}}_{k}$, k=1,…,K will estimate $E(\tilde{Y}|X_{o},Y)$ as

(4) $${\hat{P}}_{MI}=\frac{1}{K}\sum_{k=1}^{K}{\hat{P}}_{k}.$$

For full generality, the future outcomes may themselves also have missing values in the predictors, such that $\tilde{X}=\left({\tilde{X}}_{o},{\tilde{X}}_{m}\right)$, allowing that the actual missing observations may not occur in the same covariates containing missing values in the calibration data. In the presence of missing values, we will have in full generality that

(5) $$\hat{P}=E\left(\tilde{Y}|{\tilde{X}}_{o},X_{o},Y\right),$$

and similarly for the ${\hat{P}}_{k}$, which implies that the above Equation (3) should also be expanded in the obvious manner to include averaging across ${\tilde{X}}_{m}$. Furthermore, there is an additional nontrivial complication if we wish to use the predicted outcomes $\tilde{Y}$ to assess the predictive capacities of any approach in the presence of missing data ${\tilde{X}}_{m}$, as it is essential that any imputation model used for the unobserved components of $\tilde{X}$ does not make use of the associated outcomes $\tilde{Y}$. This would apply particularly for cross‐validation, but seems to generate a conflict between multiple imputation and cross‐validation, as outcomes are needed in any implementation of multiple imputation to preserve the correlation structure with the outcomes to be predicted.

## 3 PRAGMATIC IMPLEMENTATION USING EXISTING IMPUTATION SOFTWARE

In principle, implementation of the above approach is automatic and completely standard within the (fully) Bayesian approach. It has been amply described in the literature by Gelman et al. (2004), and Brooks, Gelman, Jones, and Meng (2011). Summarizing for simplicity, it consists of calibrating the conditional densities of any predictor variable, conditional on all other predictor variables and the outcome. In addition and crucially, we also need to calibrate the density of the outcome conditional on all predictors, which is the primary model component of interest in the predictive context. Missing values are treated as unknown parameters within this approach as discussed above and their estimation as well as that of any outcome proceeds in an iterative fashion starting from suitable starting values until convergence, as in regular Markov chain Monte Carlo (MCMC)‐based estimation, sequentially simulating values from the appropriate conditional densities. Optimization of this iterative sequence of equations constitutes calibration of the joint model on outcome and missing values from the primary (training) data. Once convergence is achieved, the resulting system of equations may be applied to the set of predictor values of any new observation (for which the outcome has not yet been observed) and the simulated outcome measures may be suitably summarized to generate the predicted value. The latter is essentially the approach taken in recent contributions by Erler et al. (2016) and Erler, Rizopoulos, Jaddoe, Franco, and Lesaffre (2019), for example, which is also a good recent illustration of the methodology. This approach is likely optimal from the predictive point of view. Nevertheless, it may still suffer from practical drawbacks.

1. The approach is intrinsically of much higher complexity than is customary in current traditional clinical application. Some users may have philosophical objections to the use of the fully Bayesian approach.
2. The method is difficult to implement and requires a high level of technical expertise and knowledge of Bayesian computing, which will usually be lacking.
3. The Bayesian approach may be difficult to validate, particularly in situations with small to medium sample sizes when a separate set‐aside validation set cannot be made available. This applies particulary when cross‐validation must be used.

The last is probably the most serious, besides the need to abandon the traditional Rubin multiple‐imputation compromise framework and associated software with which many researchers will be familiar. In the remainder of this paper, we restrict to cross‐validation and formulate a pragmatic approach to approximate the predictive calibration described in Section 2.2 as closely as possible using existing MI software, while also allowing for cross‐validation.

To achieve this, we first describe a general approach to validation, which allows outcome data $\tilde{Y}$ to be set‐aside for subsequent validation of prediction rules, while also allowing for the imputation of any missing data ${\tilde{X}}_{m}$ and $X_{m}$ in the corresponding validation and calibration predictor sets, respectively (Section 3.1). We then propose an algorithm that directly estimates the outcomes by pooling predictions and contrast this with an alternative approach based on direct applications of Rubin's rule (Section 3.2) for the estimation of model parameters. Although our discussion focuses on cross‐validation, it could be adapted in an obvious manner for a single set‐aside validation set.

### 3.1 Combining cross‐validation and multiple imputation

A simple approach to set‐aside outcome data and generate (multiple) imputations, while preventing the problems described in the end of Section 1 and Section 2.2, is to remove the complete set of outcomes $\tilde{Y}$ from each left‐out fold, which is defined within the cross‐validation. (Alternatively, if we have a separate validation set, we combine the validation and calibration data, after which we remove the outcomes corresponding to the validation set from this combined data set.) Imputation models may then be fit on the remainder of the observed data $\left({\tilde{X}}_{o},X_{o},Y\right)$ and imputations can be generated from these models, including for any unobserved data ${\tilde{X}}_{m}$ in the left‐out fold predictor set. In other words, the outcomes are artificially set to “missing” within the set‐aside validation fold. After imputation of the missing observations in the predictors, a suitable prediction model can be fit on the imputed calibration data $\left({\hat{X}}_{m},X_{o},Y\right)$. We then apply this model to predict the outcomes from the imputed validation predictor data $\left({\hat{\tilde{X}}}_{m},{\tilde{X}}_{o}\right)$. The outcomes $\tilde{Y}$ are then returned to the left‐out fold, after which the entire procedure can be repeated for the next fold within the entire cross‐validatory sequence. Note that the imputed values for $\tilde{Y}$ are simply discarded.

### 3.2 Combining predictive calibration with multiple imputation

With the above implementation of multiple imputation and validation, there are two basic approaches to calibrate prediction rules with multiple imputations, while allowing for cross‐validation assessment of predictions for the set‐aside outcome data with existing MI software.

The first is to define the folds on the complete data set, after which a *single imputation* and corresponding predictions for the set‐aside outcomes are generated for each fold as described above. This procedure generates a complete set of predictions across the entire data set based on application of single imputation, after which we may redefine the fold definition and repeat the procedure. In this manner, we generate a large set of cross‐validated predictions ${\hat{\tilde{Y}}}_{ik}$, across all observations i=1,…,n and for k=1,…,K for *K* repetitions of the approach. Prediction and *multiple* imputation are thus entwined in this approach and the final prediction can be derived by taking means or medians or other suitable summary, across the *K* predictions within each individual.

The second approach uses only a single fold definition, which is kept fixed across multiple imputations. For each left‐out fold in turn, *K* (multiple) imputations are then generated on the corresponding calibration and validation predictor data $\left({\tilde{X}}_{o},X_{o},Y\right)$ (with outcomes again removed from the set‐aside validation fold), after which Rubin's rule is applied to obtain estimates of the model parameters in a single consensus model. The latter single model can then be applied to generate—in principle—predictions on the *K* imputed predictor sets $\left({\hat{\tilde{X}}}_{mk},{\tilde{X}}_{o}\right)$, k=1,…,K such that we have in full generality again *K* cross‐validated predictions for each individual. The latter will of course all coincide for complete records.

**Figure 1:**
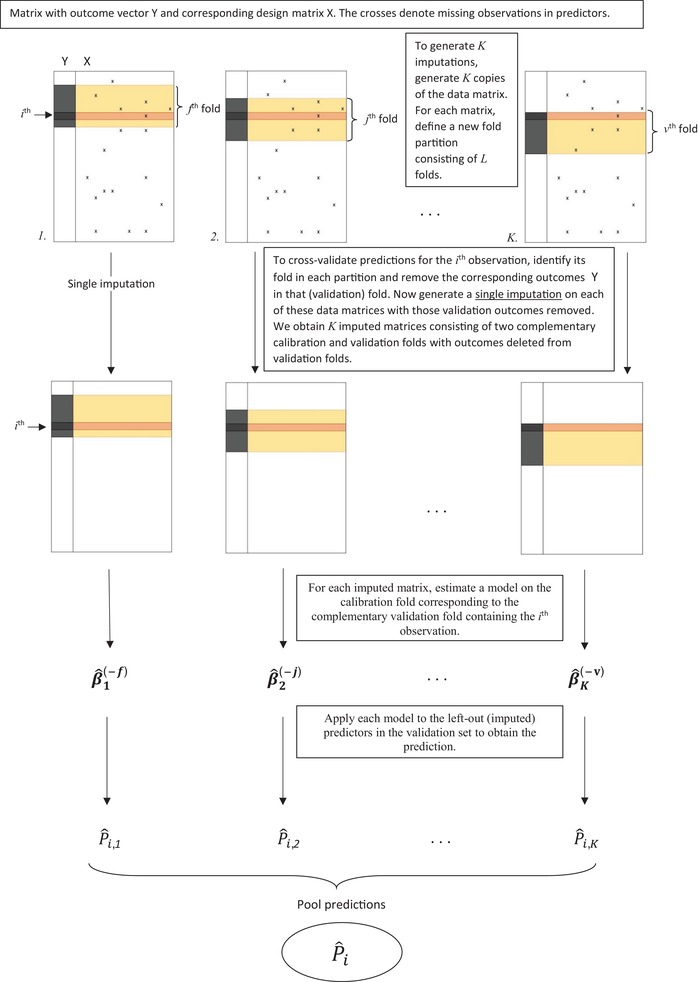
To predict outcomes through cross‐validation, Approach 1 defines *K* distinct fold partitions on *K* copies of the data‐matrix. A single imputation is then generated on the data, with outcomes deleted from the validation fold containing any *i*th observation. After imputation, the prediction model is fitted on the complementary calibration set and applied to the (potentially imputed) predictor data in the validation fold. This provides *K* predictions for each *i*th observation, which can be averaged or combined using other suitable summary measure into the final prediction ${\bar{P}}_{i}$

**Figure 2:**
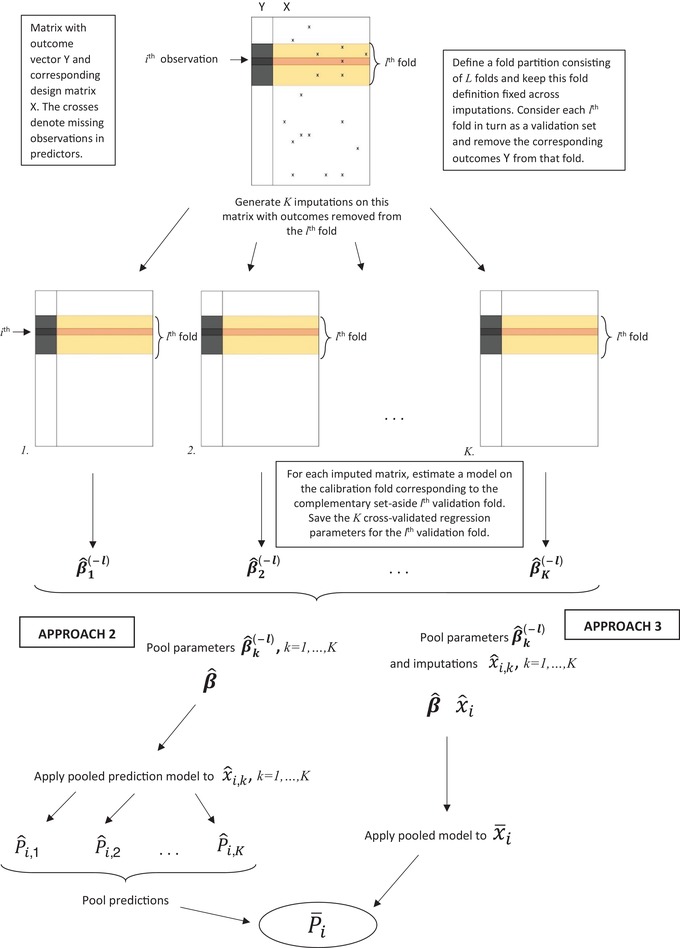
Approaches 2 and 3 use the same fold‐partition across *K* copies of the data matrix. To generate cross‐validated predictions for each (validation) fold, outcomes are first removed from that fold and *K* imputations (multiple) are performed on this outcome‐deleted matrix. *K* models are then generated on the *K* imputed calibration portions of the data, which are combined in a pooled model. This combined model is then applied to the (imputed) data in the set‐aside validation folds

A fundamental difference between the first and second approach is that we use *K* distinct models for the prediction of a single observation in the first, while there is only a single (Rubin's rule combined) model used in the second method. The other difference is the extra variation in fold definitions in the first approach. Alternatively, Approach 1 can be seen as a compromise approximation to the calibration of the predictive density as described in Section 2.2 and which can be implemented using standard software. Approach 2 on the other hand uses the model

$$f(\tilde{Y}|{\hat{\beta }}_{MI},X),$$

which is obtained by using the pooled (Rubin's rule) model parameters as plug‐in point estimators in the assumed substantive population model. Figures 1 and 2 display the structure of both approaches in the case of logistic regression with multiple imputation and cross‐validation for the analysis of binary outcome (see also the algorithmic descriptions in the supporting information). In addition to these two approaches, we also investigated a third, which is a variant of Approach 2. It consists of also averaging the imputations within the predictors (in addition to averaging the generated regression coefficients) within each individual, and then apply the pooled regression coefficient to the predictor data with missing values replaced by the averaged imputed values (Marshall et al., 2009). Code and data are implemented in an R package “*mipred*” and available on CRAN and Github (https://github.com/BartJAMertens).

We finally point out that it is implicit in the above matrix notation that different observations may have missing values in distinct covariates. Indeed, the above algorithms do not even formally require calibration and validation sets to have missing observations on the same predictors, although from a statistical point of view, different patterns of missingness could give rise to obvious concerns regarding the underlying sampling mechanisms.

## 4 DATA

We consider two data sets from our personal statistical consultation experience to illustrate and assess the proposed methodologies. Both examples investigate variation in all‐cause mortality in prospective cohort studies. The first of these (cardiac resynchronization therapy [CRT] data) studies a population subject to increased cardiovascular risk which underwent CRT to identify patients at most risk in order to improve treatment decisions. A consecutive sample of 1053 patients was collected by the Department of Cardiology of the Leiden University Medical Centre (Leiden, The Netherlands) with recruitment from August 1999 to July 2013. A Cox model was constructed using 14 predictor variables (atrioventricular junction ablation, age, gender, etiology, New York Heart Association class, diabetes, hemoglobin level, renal function, left bundle branch block, QRS duration, atrial fibrillation, left ventricular systolic [Lvdias] and diastolic functions, and mitral regurgitation); see Höke et al. (2017) for details. There were 524 patients (50%) with missing observations in predictor variables. These missing observations are furthermore almost completely concentrated in a single predictor variable (Lvdias), with negligible numbers of missing values in a restricted set of other predictors. Missing observations for Lvdias were due to failure of the measuring device. Hence, it may reasonably be assumed to represent a missing completely at random example, as missing data are caused by failure of equipment.

This does not apply to the second data set (chronic lymphocytic leukemia [CLL] data), which describes risk factors and outcomes of patients with CLL after a hematopoietic stem cell transplantation. The data were originally extracted from the registry of the European Society for Blood and Marrow Transplantation. Thirty‐two centers contributed to a data quality initiative in which these data were checked and enriched, which resulted in the data set of which an extract is presented here. This contains all 694 patients but only the variables selected in the risk factor analysis for overall survival are included in the analyses presented in the current paper (Schetelig et al., 2017a; Schetelig et al., 2017b). The predictor variables are related to the patient (age and performance status as measured by the Karnofsky Index, both at transplantation), disease (cytogenetics, remission status), previous treatment (autologous transplantation), and procedure (human leukocyte antigen [HLA] and sex match between donor and patient). The data contain 241 records with predictor missing values (35%) mainly scattered across three predictor variables. These are performance status (9% missing), remission status (6% missing), and cytogenic abnormalities (25% missing).

In this paper, we performed no variable selection and the full set of available predictors was fit (see comments in Marshall et al., 2009, on prespecification of covariates in predictive modeling, p. 2) for both the CRT and CLL data (14 and 8, respectively). For the CRT data, this corresponds to the analysis approach in the original clinical research paper (Höke et al. (2017).

To simplify the methodological and data‐analytic development, we restrict ourselves in this paper to early death within a fixed time window following patient study inclusion. This allows us to simplify to the analysis of binary outcome and logistic regression. Censored observations are treated as nonevents. For the CRT data, we consider the first two years of follow‐up, for which we have 153 deaths and 38 censored records (3.6%). For the CLL data, we only investigate one‐year survival where we have 184 early deaths and 46 censored records (6.6%).

## 5 APPLICATION AND RESULTS

We applied Approaches 1, 2, and 3 to both the CRT and CLL data. Each approach was applied using either K=1 (single imputation), 10, 100, or 1000 as number of imputations. In addition, to allow for an assessment of variation due to imputation, we repeated each application by generating 10 replicate analyses for each choice of *K*. We may then summarize the changes that occur in the cross‐validated predicted probabilities caused by changing the imputations used in the calibration, by comparing the distinct predictions between replicates. We consistently used L=10 (number of cross‐validation folds) throughout. Within any application of a method, we calculated the final cross‐validated predicted probability of the binary outcome using both the mean and the median across the *K* calibrated probabilities within an individual (note the latter will be constant by definition for patients with completely observed records [no missing predictor values] in Approaches 2 and 3). As we found very little difference between either the mean‐ or median‐based results, we decided to only present mean‐based summaries in this paper. To pool regression coefficients in Approaches 2 and 3, Rubin's rule (mean averaging) was used. Note that all approaches coincide for K=1. We emphasize that all summary performance measures are calculated on cross‐validated predictions as output by Approaches 1, 2, and 3 (Figures 1 and 2).

All analyses were carried out using R (3.4.3) (R Core team, 2017). Multiple imputations were generated using the package MICE (2.46) (van Buuren, Boshuizen, & Knook, 1999) and using chained equations and standard settings (van Buuren, 2015).

### 5.1 Summary measures

We focus on accuracy as measured by the Brier score (see Hand, 1997, section 6.5, p. 107) as well as a variance measure, which is introduced below, to compare performance between approaches on the real data.

The Brier score is calculated for each *r*th replication of an analysis with any approach for a fixed choice of *K* as

(6) $$B_{r}=\frac{1}{n}\sum_{i=1}^{n}{\left({\hat{P}}_{ir}-Y_{i}\right)}^{2},$$

with ${\hat{P}}_{ir}$ the estimated (cross‐validated) event probability for the *i*th individual in the *r*th replicated analysis and $Y_{i}$ the true class indicator. We average the 10 within‐replicate Brier scores $B_{r}$, r=1,…,10 to obtain an estimate $\bar{B}$ of the expected accuracy for the investigated approach at the number of imputations *K*.

The second summary is a measure of the amount of variation between replicate predictions ${\hat{P}}_{ir}$ for an approach with a fixed number of imputations *K* and is defined as follows. We first calculate the mean prediction ${\bar{P}}_{i}$ across replications for each patient as well as the deviations $D_{ir}={\hat{P}}_{ir}-{\bar{P}}_{i}$. While these deviations $D_{ir}$ are heteroscedastic, their variation will be *approximately* constant across patients with $0.2\leq {\bar{P}}_{i}\leq 0.8$ (Cox & Snell, 1989). We therefore discard all deviations corresponding to patients with ${\bar{P}}_{i}<0.2$ or ${\bar{P}}_{i}>0.8$ and compute the 90th and 10th percentiles *Q*_0.9_ and *Q*_0.10_ across all remaining deviations $D_{ir}$. Finally, we report $R=(Q_{0.9}-Q_{0.10})\times 100$ as a measure of spread of predictive probabilities induced by imputation variation (expressed as percentage). While this variance measure is ad hoc, it has the advantage of providing an absolute measure of the change in predicted probabilities directly at the probability scale. It is not affected by choice of transformation, such as variance stabilizing transform or the need to back‐transform to the original scale.

We calculated the above measures for both data sets and for K=1 (single imputation), 10, 100, and 1000. For the Brier score, the calculation was first carried out using all cross‐validated predictions across all patients. Then, we partitioned the set of cross‐validated predictions into the subset corresponding to patients with fully observed records (no missing data) and the subset corresponding to patients with predictors containing some missing values. We then repeated the Brier score calculation for each subset of cross‐validated predictions separately. Likewise, the variance measure *R* was calculated separately in each of both partitions (fully observed individuals and missing‐data portion). It is important to note that this is not to be confused with “complete case analysis,” as the previously described Approaches 1, 2, and 3 are always applied to the full data set. Partitioning only takes place for the calculation of the summary measures, once the derived cross‐validated predictions have been obtained from the procedures applied to the full data. We also emphasize that all results are based on cross‐validated predictions. Our reason for splitting up the summary measures calculations on partitions as described above is that, there will obviously be much more variation due to imputation for patients with missing predictors, as their variation is affected by both the direct variation in the imputed predictors, as well as the imputation variation of calculated regression coefficients. Predictions from “fully observed records” are only affected by the latter.

### 5.2 Accuracy results

**Figure 3:**
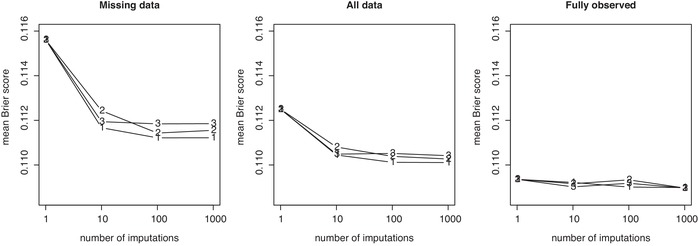
Average Brier scores for Approaches 1, 2, and 3 evaluated on the CRT data example, plotted versus the number of imputations used (K=1, 10, 100, 1000). Results are presented as calculated on the full data set (middle plot), using records containing missing values only (left‐side plot) and using the complete cases only (right‐side plot), but always based on models calibrated on the full data. The plotting symbol (1, 2, or 3) indicates the approach used

**Figure 4:**
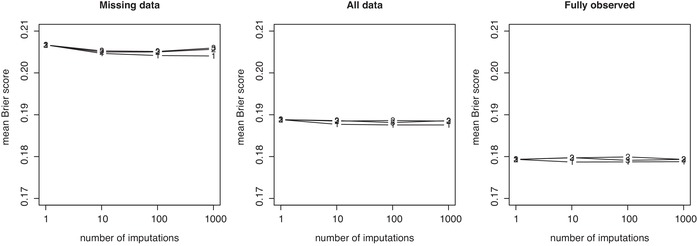
Average Brier scores for Approaches 1, 2, and 3 evaluated on the CLL data, plotted versus the number of imputations used (K=1, 10, 100, 1000). Results are presented as calculated on the full data set (middle plot), using records containing missing values only (left‐side plot) and using the complete cases only (right‐side plot), but always based on models calibrated on the full data. The plotting symbol (1, 2, or 3) indicates the approach used

Figures 3 and 4 display results for Brier scores. The different plotting symbols 1, 2, and 3 distinguish between the three approaches. As expected, Brier scores are always higher when calculated on records containing missing values, due to the greater uncertainty induced through the need to estimate these in imputation. Results calculated from the complete data are a compromise between Brier scores on the fully observed cases and those for records containing missing data.

Crucially, for accuracy, results do not seem to differ between the approaches, whether investigating the CRT or CLL data. For the CRT data, we notice a small decrease in Brier scores from K=1 to K=10 in both the missing data and when calculated across the entire data set. The same effect cannot be seen in the fully observed part of the data, which indicates that the slight gain in accuracy is due to the increased precision gained by multiple as opposed to single imputation. There does not seem to be further gain when increasing imputations beyond K=10, however. In comparison and for the CLL data, Brier scores are essentially constant across *K*.

### 5.3 Variation results

**Figure 5:**
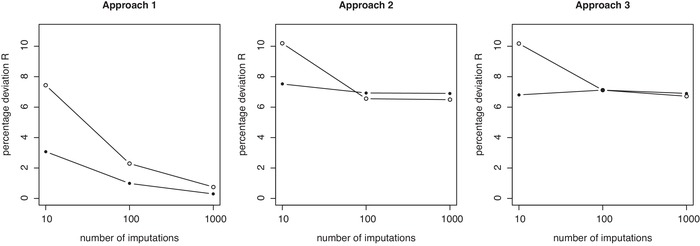
Percentage deviations of predictions *R* across replicate calibrations for Approaches 1, 2, and 3 in the CRT data example, plotted versus the number of imputations used (K=10, 100, 1000). Results are shown separately for fully observed records (solid dots) and observations containing missing observations (open dots), but always based on models calibrated on the full data. *R* measures at K=1 are 9.9% for fully observed records and 20.6% for missing observations and identical across approaches (hence not shown in above plots)

**Figure 6:**
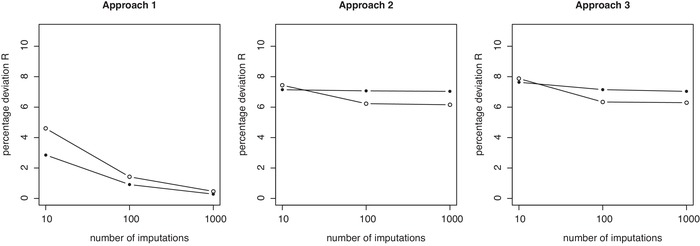
Percentage deviations of predictions *R* across replicate calibrations for Approaches 1, 2, and 3 in the CLL data example, plotted versus the number of imputations used (K=10, 100, 1000). Results are shown separately for fully observed records (solid dots) and observations containing missing observations (open dots), but always based on models calibrated on the full data. *R* measures at K=1 are 9.6% for fully observed records and 15.3% for missing observations and identical across approaches (hence not shown in above plots)

Calculating the variation measure *R* for K=1, corresponding to single imputation and for which all three approaches coincide, gives R=20.6% and R=9.9% when summarizing predictions in the partially and fully observed records, respectively, in the CRT data. For the CLL data, these numbers are R=15.3% and R=9.6% for partially and fully observed records, respectively. As expected, predicting from fully observed records is “more easy” in the sense that it is associated with less variability, which is a consistent feature of the full results for K=10,100,100 with the first approach as shown in Figures 5 and 6. It is due to prediction for fully observed records not being affected by the variation induced by the need to estimate the unobserved predictors using imputation, as for the partially observed records, in addition to the variation in regression coefficients induced by imputation. The most striking feature may however be the magnitude of the absolute deviations among predicted probabilities fitted for K=1, due to imputation variation.

Figures 5 and 6 show the change in the variation measure *R* when increasing *K*. The behavior is very different between Approach 1 and Approaches 2 and 3. For the CRT data and Approach 1, increasing *K* to 10 imputations leads to a reduction of the variation measure to 7.4% and 3.1% for partially and fully observed data, respectively. These numbers gradually further decrease as we increase *K* to 100 and 1000. Specifically *R* reduces from 7.4%, to 2.3% and 0.8% for partially observed data. For fully observed data, the reduction is from 3.1% to 0.9% to 0.3%.

We can note how the variation measures reduce similarly for Approaches 2 and 3 with increasing *K*, but very differently from Approach 1. First note how an increase to K=10 only reduces *R* to 10.1% and 7.5% for Approach 2. Further reductions as we increase to K=100 and 1000 are much smaller, as we have R= 6.6% and 6.5% for partially observed records at 100 and 1000 imputations, respectively. Similarly we have R= 6.9% and 6.9% for fully observed records at 100 and 1000 imputations. Results from Approach 3 are virtually identical.

Only for Approach 1 do we observe a gradual decrease in variation of predictions as *K* increases and as one would reasonably expect. For Approaches 2 and 3, the gains are however much smaller and nonexistent once we have reached K=100, after which no further reductions in variation are observed. For any given level of *K*, Approach 1 beats Approaches 2 and 3 in terms of variation and for both fully and partially observed data. Note how the variation measures at K=1000 for Approaches 2 and 3 are barely improving on the variation we can observe at K=10 for Approach 1 already.

For Approach 1, we can note that the payoffs for increased imputation face diminishing returns, although the variation continues to reduce toward the zero lower bound. It is of interest that only for K=1000 and Approach 1, variation is reduced to levels which may be acceptable for clinical application.

Results from the analysis of the CLL data (Figure 6) are from a qualitative point of view a complete confirmation of the above observations. At K=10 and Approach 1, for example, R= 4.6% and 2.9% for partially and fully observed records, with further reductions for increasing *K* more modest but with variation gradually approaching zero. For Approaches 2 and 3, these numbers are 7.4% and 7.1% (Approach 2) and 7.8% and 7.6% (Approach 3) and with negligible further reductions as *K* is increased to 1000. In fact, for the CLL data, variation measures *R* are completely separated between Approach 1 on the one hand and Approaches 2 and 3 on the other. The lowest variation measure R=6.2 at K=1000 for Approach 2 on partially observed records, is substantially above R=4.6% for partially observed data for Approach 1 with K=10. Again, we only achieve variation levels for *R* of 0.5% and 0.3% at K=1000 for Approach 1, which again indicates that imputation numbers may need to be substantially increased beyond current practice.

Finally concerning Approach 3, we note that neither gain nor loss of performance is observed relative to Approach 2 in terms of accuracy and variance. Importantly, however, this also implies that using the mean imputation in prediction does not reduce the performance deficit relative to Approach 1.

### 5.4 Simulation

We use simulations to assess differences between methodologies in terms of mean square error (Brier score) and variation, but also bias, which cannot be assessed in data‐based analyses. Simulations are inspired by the variance–covariance structures observed in the CRT data. We simulated 100 data sets of 1000 observations each and containing a univariate binary outcome measure *Y* as well as a four‐dimensional predictor vector $X=(X_{1},\ldots ,X_{4})$ for each observation. To simulate each data set, 1000 observations are first drawn for X from the multivariate normal $N_{4}\left(\mu ,\Sigma \right)$, with μ=0 and Σ set equal to the observed variance–covariance matrix of the four continuous covariates in the CRT data set. Binary outcomes are generated from the Bernoulli distribution with probability *P*, determined by a logistic regression model $\ln\left(P/\left(1-P\right)\right)=\beta_{0}+X_{1}\beta_{1}+\cdots +X_{4}\beta_{4}$. Missing values are then introduced for *X*_1_, either completely at random (MCAR) or at random (MAR). For the MAR scenario, observations in *X*_1_ are set to missing, based on the simulated binary variable (missing yes/no) from a Bernoulli distribution with probability $U_{i}=\min\left[X_{2i}^{*}\;M/\;{\bar{X}}_{2}^{*},1\right]$ for each *i*th observation, with *M* a constant to control the number of missing values and $X_{2i}^{*}=\left(X_{2i}-\min\left(X_{2}\right)\right)/\left(\max\left(X_{2}\right)-\min\left(X_{2}\right)\right)$.

**Figure 7:**
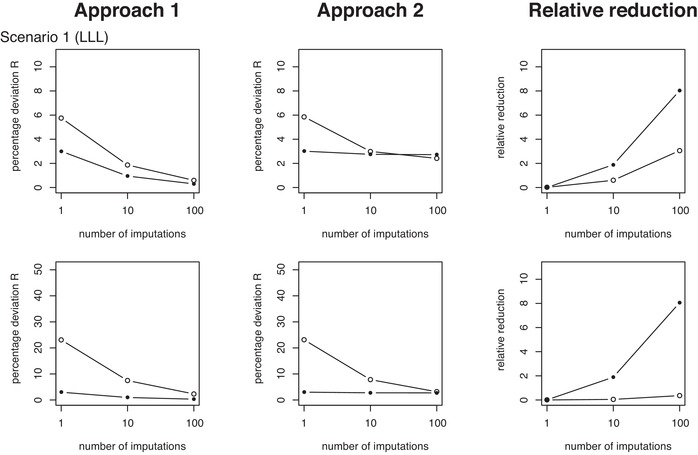
Measures of average percentage prediction deviations ($\bar{R}$). The two rows of plots from top to bottom correspond to simulation MCAR Scenarios 1 and 3. The two left columns of plots show results from Approaches 1 and 2 (as also shown in Figures 5 and 6) versus the number of imputations used in the calibration of the predictors. The right‐side column of plots displays the corresponding relative variance reductions for Approach 1 relative to Approach 2. See Table 1 for description of the scenarios. Results for all other scenarios and MAR are found in the supporting information

Simulations are generated with the above setup for eight scenarios in a 2^3^ design corresponding to all combinations of the following three binary options. Set $\beta_{0}=-1.39$ or $\beta_{0}=0.405$, corresponding to either P(1|X=0)=0.2 or P(1|X=0)=0.6. Put either $\beta_{1}=\log\left(1.1\right)$ or $\beta_{1}=\log\left(2\right)$, keeping the other regression effects fixed for all simulations as $\beta_{2}=\log\left(2\right)$, $\beta_{3}=\log\left(0.85\right)$, and $\beta_{4}=\log\left(0.75\right)$. Set *M* such that we obtain either 10% or 50% missing values for *X*_1_. Table 1 summarizes the scenarios and provides the shorthand reference notation used in Figure 7 as well as in the full set of tables and figures in the supporting information.

**Table 1:** Summary description of the simulation scenarios investigated. A 2^3^ design is used, corresponding to all combinations of either Low (L) or High (H) for the parameters β_0_, β_1_, and the percentage of missing values. These scenarios are investigated in either the MCAR or MAR case. See also Figure 7 and supporting information for variance results ($\bar{R}$ measure) on these scenarios in the MCAR case

Scenario	β_0_	β_1_	% Missing	Shorthand Reference Notation
1	−1.39	log (1.1)	10%	LLL
2	0.405	log (1.1)	10%	HLL
3	−1.39	log (2)	10%	LHL
4	0.405	log (2)	10%	HHL
5	−1.39	log (1.1)	50%	LLH
6	0.405	log (1.1)	50%	HLH
7	−1.39	log (2)	50%	LHH
8	0.405	log (2)	50%	HHH

For each of the above eight scenarios, cross‐validated predictions were generated with Approaches 1 and 2, using the previously described algorithms and for either K=1, 10, or 100 imputations. For each data set generated, each analysis was replicated 10 times (using a completely new set of imputations) to assess the impact of imputation variation on the predicted probabilities. The previously described average Brier score $\bar{B}$ and variance measure *R* were calculated. Bias is similarly defined for each *r*th replication as

$$Bias_{r}=\frac{1}{n}\sum_{i=1}^{n}\left({\hat{P}}_{ir}-P_{i}\right),$$

with ${\hat{P}}_{ir}$ the fitted and $P_{i}$ the simulated true probability for the *i*th individual. This measure is then averaged over all replicates within each simulated data set to obtain the final average bias measure $\bar{Bias}$. Finally, this procedure is repeated for 100 distinct data sets generated from the above simulation setup and the averages are taken of all summary measures for Brier score ($\bar{\bar{B}}$), variance ($\bar{R}$), and bias ($\bar{\bar{Bias}}$) across all data sets for each scenario.

In brief, the simulations confirm and further support our findings from data analysis. Figure 7 shows results on the variance measure $\bar{R}$ for simulation scenarios 1 (LLL) and 3 (LHL) in the MCAR case and for K=1, 10, and 100 imputations, respectively, with similar setup as for the CRT and CLL data analyses (Figures 5 and 6). The full set of figures and table summaries for MCAR as well as MAR across all scenarios (1–8) can be found in the supporting information. In these figures, each row of plots corresponds to a single scenario, with the left‐most and middle plots displaying results from Approaches 1 and 2, respectively. The plots to the right show the relative reductions $\left({\bar{R}}_{2}-{\bar{R}}_{1}\right)/{\bar{R}}_{1}$ of the variance summary measure between Approaches 2 and 1, with ${\bar{R}}_{2}$ and ${\bar{R}}_{1}$ the measures from Methods 2 and 1, respectively. The similarity of variance results from Figure 7 (and likewise for figures in the supporting information) with the data analytic results on the CRT and CLL examples in Figures 5 and 6 is clear. The key difference between Scenarios 1 and 3 is the much higher variances when the effect size β_1_ is large (Scenario 3). The same is observed in the supplementary results for Scenarios 4, 7, and 8 versus all other scenarios (1, 2, 5, 6). Otherwise, variance patterns are similar from qualitative point of view. Results from the MAR case are virtually identical to those from MCAR and can be found in the supporting information.

The supporting information contains tables for all statistics, summarizing the variance, Brier, and bias measures for both the MCAR and MAR scenarios and K=1, 10, and 100 imputations, respectively, with Monte Carlo standard errors (MC *SE*s). The previously described levels of the simulation parameters β_0_, β_1_, and *M* are denoted in these tables as either low (L) or high (H) (see also Table 1). In addition, the supporting information displays figures of these tabulated results for the bias and Brier score measures discussed above across all scenarios. These figures also show relative reductions for Brier score and bias measures between approaches, with compatible definition to the above for variance.

The key results are again found in the systematically and substantially lower levels of variation observed for the first approach (A1 in the tables) across all scenarios investigated, as compared to the second approach (A2), although the absolute levels of variation are less extreme than observed in the real data. Variation tends to be lower when predicting from fully observed records as compared to prediction with records containing missing data, but again this applies particularly to the first approach. Reduced variation for prediction of fully observed records is not observed as consistently for Approach 2 and the variance reductions are also smaller. Note how the relative differences between Approaches 1 and 2 increase with increased numbers of imputations, in favor of Approach 1. We did not find evidence of important differences between approaches in Brier scores or bias measures for any scenario. In particular, the relative reduction measures between Approaches 1 and 2 for Brier score and bias are effectively zero for all scenarios and irrespective of the number of imputations used. Biases tend to be small, except for simulations with β_1_ large and when records have partially missing predictors. For these scenarios, we also observed small reductions in Brier scores when predicting on fully observed records as compared to partially missing records.

## 6 CONCLUSIONS AND RECOMMENDATIONS

We have investigated the problem of combining predictive calibration with (cross) validation in prognostic applications, when multiple imputations are used to account for missing values in predictor data. Instead of following a primarily algorithmic ad hoc approach, we have commenced with a methodological review of multiple imputation in the predictive setting. Specifically, we clarified how predictive calibration requires estimation of a different predictive density (Equation (3))—and thus integration across both missing observations and unknown effect parameters—as opposed to averaging across missing values only (Equation (1)), which is implicitly implemented in current standard multiple imputation software. Instead of pursuing a direct, fully Bayesian approach to the calculation of the integrals as in Erler et al. (2016) and Erler et al. (2019), we have proposed a methodology that estimates by approximation the expectation of the required predictive density. We achieve this by averaging the predictions from individual models fitted on the single imputed data sets within a set of (multiple) imputations that can be generated with existing multiple imputation software (Approach 1). We contrasted this methodology with direct use of Rubin's rules‐based model calibrations (Approaches 2 and 3). Finally, we compared methods on accuracy and variance measures calculated on cross‐validated estimates of the predicted probabilities in two real data sets, and in a simulation study.

Results suggest that methodological approaches are indistinguishable with respect to accuracy (Brier score). Large differences from both the qualitative and quantitative point of view are however observed between Approach 1 (combining predictions) and Approaches 2 and 3 (pooling regression coefficients), with respect to the variation of predictions for individual patients between repetitions of the procedure with different imputations (variability due to imputation variation). The following observations can be made:

1. Absolute levels of variation of predicted probabilities due to imputation variability are very high when predictions are based on models calibrated using a single imputation only.
2. Use of multiple imputations reduces this variation, but Approach 1 is vastly more efficient at reducing variation of predicted probabilities as compared to Approaches 2 and 3, for the same increase in imputation numbers.
3. Only Approach 1 appears to have the basic property of variation reducing to zero as the number of imputations increases. For Approaches 2 and 3, variance measures stabilize once 100 imputations have been used and do not reduce further.

From these observations, the following general recommendations can be formulated on the use of imputation to account for missing values, when the primary objective is to predict (binary) outcome in future patients, based on models calibrated on previously observed data from the same population.

1. Multiple imputations must be used to reduce the variation of predicted probabilities which is due to imputation. Use of single imputation in predictive calibration should be rejected and the practice phased out, irrespective of the prediction methodology chosen when imputation is applied.
2. One should use direct averages or other suitable combination of the individual predictions (Approach 1), obtained from the models fitted on the individual imputed data sets within a set of multiple imputations, instead of application of a single combined model for prediction, such as given by the Rubin's rules pooled model (Approaches 2 and 3), for example.
3. Numbers of imputations used in predictive modeling must be drastically augmented above current practice to reduce variation to levels suitable for routine clinical application. While numbers closer to 1000 imputations or beyond may be needed, this issue requires further research and dedicated procedures to estimate imputation numbers in specific applications. A literature review (Marshall et al., 2009, p. 6) indicates the majority of clinical applications used between 5 and 10 imputations.

We emphasize that the results from this paper in favor of Approach 1 are relevant when applying statistical models specifically for the primary purpose of prediction. If the objective is instead to learn about the nature of the dependence of outcome on predictors, as in etiologic research, for example, then the findings described in this paper are not relevant. Put simply, if we are only interested in reporting effect estimates and their standard errors, we continue to use the classical results and methods on application of Rubin's rules for pooling of multiple models described in existing literature (Carpenter & Kenward, 2013; Carlin, 2015). Conversely however, even when we are specifically interested in calibration of a predictive rule, this does not imply that the Rubin's rules pooled model suddenly becomes uninteresting or irrelevant. Indeed, even though we should use averages or other combinations of predictions from distinct models on multiple imputations as described in this paper, we may still wish to report information about the extent to which the constituent prediction models that contribute to this combined prediction vary between one another across the imputations. Likewise, we would like to get a sense of the “average model.” The Rubin's rules pooled model and standard errors are well suited for this purpose. This information may continue to be reported in applied papers, side‐to‐side with summary measures on the predictive potential of the combined predictor.

## 7 DISCUSSION

Irrespective of the above results, we hope our paper would stimulate the medical statistical community to propose future work on a combination of multiple imputation, predictive calibration, and validation by reference to the methodological outline described in Section 2 of this paper. In principle, pursuing a fully Bayesian approach would ensure such rigor as it automatically leads to calibration of the required integrals described in Section 2. The recent papers by Erler et al. (2016, 2019) are an example of this approach, although specifically concerned with the analysis of repeatedly observed longitudinal outcome data and thus outside the scope of our contribution. Indeed, our proposed methodology (Approach 1) may be viewed as an approximation of such fully Bayesian implementations with existing software. An alternative to Bayesian modeling might be to adapt existing multiple imputation software such that it allows to save the imputation model equations for use in the imputation of future observations, which may have missing predictor values.

Ideally novel software should facilitate modeling of the substantive outcome to be predicted, such that both objectives (imputation and predictive modeling) can be achieved simultaneously. To our knowledge, current multiple imputation software is restricted to estimation of (pooled) regression (effect) measures and standard errors within a fixed data set. We should also point out that, with exception for the analysis of continuously distributed data using the joint multivariate normal (Hughes et al., 2014) and again as far as we are aware, the MICE software on which the implementation of approaches described in this paper relies on (as for similar imputation software in STATA or SPSS, for example) has no solid theoretical basis, and specifically cannot be interpreted as a formal “Gibbs sampling” implementation. This of course in turn also constrains the theoretical underpinning of the implementations of approaches compared in this paper, which are based on MICE.

### 7.1 Imputation, variance, and bias

In the next few paragraphs, we highlight specific issues where our paper makes contributions to current literature. First, our work has reviewed imputation methodology and its connection with predictive density calibration, which may be viewed as underlying ideas present in a more rudimentary manner in the paper by Miles (2015), in addition to an extensive practical assessment. The paper by Miles concludes with a more equivocal assessment between predictive approaches, which is however largely due to the exclusive focus on mean square prediction error. It is remarkable that current literature on multiple imputation in prediction applications ignores the impact of the large variability associated with imputation. An important contribution of the present paper is to show that this imputation variation affects the resulting prediction in very different ways between the approaches studied with prediction averaging the clear winner. From the predictive point of view and when imputation is used, variation may be at least as important as potential bias, if not more. Indeed, trading variance for bias is a successful prediction calibration strategy utilized in shrinkage estimation, for example (Hastie, Tibshirani, & Friedman, 2008). To stress the importance of variation when studying predictive calibration with imputations is consistent with the focus on variation due to imputation in the existing literature concerned with application of multiple imputation for effect estimation. We hope that future work will have greater attention for the assessment of predictive variation.

### 7.2 Imputation, predictive performance assessment, and validation

The (cross) validatory implementations proposed in our paper avoid the inherent bias caused by using the same data for both model fit and the presented predictive assessments. Failure to do so is a persistent weakness that invalidates comparisons made and presented in current literature, including the papers by Miles (2015), Wood et al. (2015), and even the contribution from Wahl et al. (2016). If one only wishes to assess prognostic performance, then an alternative approach discussed by some authors is to directly apply Rubin's rules to measures of “model fit and performance,” calculated on models fit to single imputations, as described in (Marshall et al., 2009, see p. 3 of the paper), but the idea is also implicit in Wood et al. (2015). To discuss the relative merits of this procedure to those investigated in this paper, we need to keep the central objective and paradigm studied in this paper in mind. This is to investigate, in the context of missing values and imputation, the problem of how to calibrate predictive methods on the one hand—and how to assess them on the other. The fundamental process used in the paper is that any method eventually used for prediction should be specified before application to the prediction of new outcome data, such that any predictive performance measure can be calculated on predictions obtained from application of such models and may thus be unambiguously ascribed to that specific prediction method. This paradigm is an established principle and should not be put in dispute, even when investigating missing data problems. The paper achieves this objective by considering various formulations of how predictive rules could be calibrated in the presence of missing data—one being to plug in pooled estimates of unknown parameters calculated using distinct numbers of imputations for missing values—the other an approach that integrates out the missing values by approximation using distinct numbers of imputations. The paper implements these methods for various choices of the numbers of imputations and then applies them to outcome data that have not previously contributed to the calibration of the models to obtain the performance measures. As such, we adhere to basic principles of model building and subsequent assessment.

A Rubin's rule average of performance estimates from individual prediction models, each fitted on a single imputation, might be viewed as an estimate in the above described sense, as what could be expected on average from a prediction model estimated on a single imputation, with the standard deviation giving a measure of uncertainty due to changes in the individual predictions from one (single imputation–based) model to the next. As the current paper demonstrates, however, this procedure unfortunately does not generally give the performance estimates, with the above interpretation, for either the Rubin's rule pooled model, or for the prediction‐averaging approach (see Figures 5 and 6, as well as simulation results). An advantage of direct application of Rubin's rules pooling of performance estimates is that it can give estimates of standard errors on the pooled summary performance estimate. Easy availability of a standard error is however no guarantee of the suitability of a statistic as an estimator. Standard error calculation of summary performance estimates for the prediction methods discussed in this paper could be achieved in principle through error propagation. Methodologies to implement this would need to be developed and themselves validated.

### 7.3 Imputation for prediction and machine learning

The methodology presented in this paper explicitly allows for and accommodates the imputation of missing data in the predictors of new observations to be predicted (as explained Section 3.1). The recent paper by Mercaldo and Blume (2018) is relevant in this context for the interesting introductory overview on current approaches to imputation in prediction (Section 1.2). Indeed, authors state that in imputation‐based approaches “… the additional out‐of‐sample record should be combined with the original data, and the full imputation algorithm should be refit to properly fill in missing values.” Obviously, this is a complicating aspect of imputation‐based solutions and difficult to achieve, but it is precisely this approach that is fully implemented and evaluated in our paper. An alternative, if we wish to avoid this difficulty, is to avoid imputation altogether by calibrating multiple prediction scores for distinct missingness patterns in the data and then only apply those scores that are compatible with the fully observed data portion of the new record to be predicted. The Mercaldo and Blume paper investigates this approach extensively. The practice of augmenting the original calibration data with the out‐of‐sample data in imputation for practical prediction application requires further study, which we hope to carry out in future research and publication.

We should point to an obvious connection between machine learning, particularly ensemble learning, and Approach 1. It is known that ensemble methods, for example, Breiman (1996), Wolpert (1992), and (Hastie et al., 2008, section 8.8), can be highly effective at variance reduction through averaging of predictions from multiple constituent models. The latter are usually obtained through refitting of some base model, often after perturbation of the data in some sense, such as bootstrapping. In our case, the perturbations can be thought of as arising from distinct realizations of the required imputation. Breiman (1996) explains how averaging methods, as for Approach 1, can produce large predictive gains when “instable” predictors are combined. In connection to machine learning methodology, it is also of interest to note that Approach 1 is generally applicable across all prediction methods, irrespective of whether these have a formal statistical methodological foundation, whereas Approach 2 can only be applied to the subclass of prediction models for which Rubin's rule pooling of model coefficients can be applied.

### 7.4 Data‐based evaluation, implementation, and relevance

Finally, we hope our paper would stimulate the medical statistical community to evaluate model approaches on real data and to study data‐based summary statistics such as accuracy or direct measures of variance as in this paper—and not solely rely on simulations that can too easily be adapted or selected to suit researchers' needs or preconceived ideas. Current literature on predictive calibration and validation with imputation typically reverts to simulations‐only evaluations, sometimes presented as “data‐based” simulations. See also Boulesteix et al. (2018) for a recent discussion on improving simulation studies.

We conclude with two remarks on implementation and relevance. First, we have consistently used means to pool the predicted probabilities (as for the regression coefficients using Rubin's rule) in this paper. One could however imagine other choices, such as averaging at the logit scale, use of medians, and so on, which would not substantially alter the nature of the approaches shown. For the research in this paper, we have recalculated all results using medians and found results that are both from a quantitative, and hence also qualitative, point of view near identical to the results shown here. We also visually inspected the distributions of probabilities averaged and found them to be near‐symmetric near the mode. To simplify the presentation, we therefore decided to use the mean throughout.

Our paper has focused on logistic regression for binary outcome in prognostic studies. We have already pointed out that our results and conclusions are likely to apply to prediction problems with generic outcome and should thus be viewed as of general relevance to the statistical literature on prediction and are unlikely to restrict to binary outcome only. Likewise, we anticipate that similar conclusions will apply to models beyond logistic regression studied here. For prognostic studies and to achieve full generality, the extension to life‐time outcomes in the presence of censoring should also be investigated, particularly for Cox models. This entails some special complications apart from censoring, particularly the need to also address variation in baseline hazards as well as special considerations as to how censored survival outcomes should be accounted for within multiple imputation (Carpenter & Kenward, 2013, chapter 8). We have executed this research and based on this can confirm that the key results from the present paper on mean square error and variance for binary outcome do indeed carry over to the survival setting with Cox regression analysis. The description of this research however requires a separate dedicated paper.

## CONFLICT OF INTEREST

The authors have declared no conflict of interest.

## Supporting information

SUPPORTING INFORMATION

SUPPORTING INFORMATION

## References

[bimj2104-bib-0001] Boulesteix, A.‐L. , Binder, H. , Abrahamowicz, M. , & Sauerbrei, W. , for the Simulation Panel of the STRATOS Initiative (2018). On the necessity and design of studies comparing statistical methods. Biometrical Journal, 60(1), 216–218. 10.1002/bimj.201700129 29193206

[bimj2104-bib-0002] Breiman, L. (1996). Bagging predictors. Machine Learning, 24(2), 123–140.

[bimj2104-bib-0003] Brooks, S. , Gelman, A. , Jones, G. , & Meng, X.‐L. (2011). Handbook of Markov chain Monte Carlo. New York: NY: Chapman and Hall/CRC.

[bimj2104-bib-0004] van Buuren, S. (2015). Fully conditional specification In Molenberghs G., Fitzmaurice G., Kenward M., Tsiatis A., Verbeke G. (Eds.), Handbook of missing data methodology. Boca Raton, FL: Chapman and Hall.

[bimj2104-bib-0005] van Buuren, S. , Boshuizen, H. , & Knook, D . (1999). Multiple imputation by chained equations in R. Journal of Statistical Software, 45, 1–67.

[bimj2104-bib-0006] Carlin, B. (2015). Multiple imputation: A perspective and historical overview In Molenberghs G., Fitzmaurice G., Kenward M., Tsiatis A., Verbeke G. (Eds.), Handbook of missing data methodology. Boca Raton, FL: Chapman and Hall.

[bimj2104-bib-0007] Carpenter, J. , & Kenward, M. (2013). Multiple imputation and its application. New York: Wiley.

[bimj2104-bib-0008] Cox, D. R. , & Snell, E. J. (1989). Analysis of binary data. New York: Chapman and Hall.

[bimj2104-bib-0009] Erler, N. , Rizopoulos, D. , Jaddoe, V. , Franco, O. , & Lesaffre, E. (2019). Bayesian imputation of time‐varying covariates in linear mixed models. Statistics Methods in Medical Research, 28(2), 555–568. 10.1177/0962280217730851 PMC6344996 29069967

[bimj2104-bib-0010] Erler, N. , Rizopoulos, D. , van Rosmalen, J. , Jaddoe, V. , Franco, O. , & Lesaffre, E . (2016). Dealing with missing covariates in epidemiological studies: A comparison between multiple imputation and a full Bayesian approach. Statistics in Medicine, 35, 2955–2974. 27042954 10.1002/sim.6944

[bimj2104-bib-0011] Gelman, A. , Carlin, J. , Stern, H. , & Rubin, D. (2004). Bayesian data analysis. Boca Raton, FL: Chapman and Hall/CRC.

[bimj2104-bib-0012] Hand, D. J. (1997). Construction and assessment of classification rules. Chichester, West Sussex: Wiley.

[bimj2104-bib-0013] Hastie, T. , Tibshirani, R. , & Friedman, J. (2008). The elements of statistical learning. New York: Springer‐Verlag.

[bimj2104-bib-0014] Höke, U. , Mertens, B. , Khidir, M. , Schalij, M. , Bax, J. , Delgado, V. , & Marsan, N. (2017). Usefulness of the CRT‐SCORE for shared decision making in cardiac resynchronization therapy in patients with a left ventricular ejection fraction of ⩽35. American Journal of Cardiology, 120(11), 2008–2016. 29031415 10.1016/j.amjcard.2017.08.019

[bimj2104-bib-0015] Hughes, R. , White, I. R. , Seaman, S. R. , Carpenter, J. R. , Tilling, K. , & Sterne, J. A. C . (2012). Joint modelling rationale for chained equations imputation. BMC Medical Research Methodology, 14, 28. 10.1186/1471-2288-14-28 PMC3936896 24559129

[bimj2104-bib-0016] Lessafre, E. (2012). Bayesian biostatistics. New York, NY: Wiley.

[bimj2104-bib-0017] Marshall, A. , Altman, D. , Holder, R. , & Royston, P. (2009). Combining estimates of interest in prognostic modelling studies after multiple imputation: Current practice and guidelines. BMC Medical Research Methodology, 9, 57, 10.1186/1471-2288-9-57. 19638200 PMC2727536

[bimj2104-bib-0018] Mercaldo, S. F. , & Blume, J. D. (2018). Missing data and prediction: The pattern submodel. Biostatistics, 10.1093/biostatistics/kxy040. PMC7868046 30203058

[bimj2104-bib-0019] Miles, A. (2015). Obtaining predictions from models fit to multiply imputed data. Sociological Methods and Research, 45(1), 175–185.

[bimj2104-bib-0020] R Core Team . (2017). R: A language and environment for statistical computing. Vienna: R Foundation for Statistical computing.

[bimj2104-bib-0021] Schetelig, J. , de Wreede, L. C. , van Gelder, M. , Andersen, N. S. , Moreno, C. , Vitek, A. , … Kröger, N . (2017a). Risk factors for treatment failure after allogeneic transplantation of patients with CLL: A report from the European Society for Blood and Marrow Transplantation. Bone Marrow Transplantation, 52, 552–560. 28112746 10.1038/bmt.2016.329

[bimj2104-bib-0022] Schetelig, J. , de Wreede, L. C. , Andersen, N. S. , Moreno, C. , van Gelder, M. , Vitek, A. , … Kröger, N. , CLL subcommittee, Chronic Malignancies Working Party . (2017b). Centre characteristics and procedure‐related factors have an impact on outcomes of allogeneic transplantation for patients with CLL: A retrospective analysis from the European Society for Blood and Marrow Transplantation (EBMT). British Journal of Haematology, 178, 521–533. 28589551 10.1111/bjh.14791

[bimj2104-bib-0023] Seber, G. A. F. (1984). Multivariate observations. New York, NY: Wiley.

[bimj2104-bib-0024] Vergouwe, Y. , Royston, P. , Moons, K. , & Altman, D . (2010). Development and validation of a prediction model with missing predictor data: A practical approach. Journal of Clinical Epidemiology, 63, 205–214. 19596181 10.1016/j.jclinepi.2009.03.017

[bimj2104-bib-0025] Wahl, S. , Boulesteix, A.‐L. , Zierer, A. , Thorand, B. , & van de Wiel, M. (2016). Assessment of predictive performance in incomplete data by combining internal validation and multiple imputation. BMC Medical Research Methodology, 16(1), 144. 27782817 10.1186/s12874-016-0239-7 PMC5080703

[bimj2104-bib-0026] Wolpert, D. (1992). Stacked generalization. Neural Networks, 5(2), 241–259.

[bimj2104-bib-0027] Wood, A. , Royston, P. , & White, I . (2015). The estimation and use of predictions for the assessment of model performance using large samples with multiply imputed data. Biometrical Journal, 57, 614–632. 25630926 10.1002/bimj.201400004 PMC4515100

