# Supplementary material for: Construction and assessment of prediction rules for binary outcome in the presence of missing predictor data using multiple imputation and cross‐validation: Methodological approach and data‐based evaluation
Source: Biom J. 2020 Feb 13;62(3):724–41. doi: 10.1002/bimj.201800289 (PMC7217034; doi:10.1002/bimj.201800289)
Supplement: Supplementary file 1 — SUPPORTING INFORMATION [file BIMJ-62-724-s002.pdf]

1 Simulation study

1.1 Description of simulation set-up

- 100 datasets with each 1000 individuals were simulated.
- For each individual, four covariates (X_j) were drawn from $N_4(\mu, \Sigma)$, with $\mu_j = 0$ and Σ equal to the variance-covariance matrix of four continuous covariates in the CRT dataset.
- For each individual, a binary outcome was generated by means of a draw from a binomial distribution with probability P, where P was determined in a logistic regression model $\ln(P/(1-P)) = \beta_0 + X_1\beta_1 + ... + X_4\beta_4$ in which $X_1, \ldots, X_4$ were the predictors.
- Missing values were then introduced into X_1, either according to a MCAR mechanism or a MAR mechanism. For MAR, the probability of being missing depended on the value of X_2 (see paper for the precise definition). The number of missing values generated is governed by a parameter M which is set to either a low or high value to generate less or more missing values in X_1.
- Three parameters were varied as also explained in the paper, between either a low (L) or set to a high level (H). The tables below (and in the paper) use the same notation L/H to denote the scenarios as all 8 combinations of these 3 binary settings. The approaches used are indicated in the tables as A1 for approach 1 and A2 for approach 2.
  - $\beta_0 = -1.39$ (L) or $\beta_0 = 0.405$ (H), corresponding to $P(1|X = 0) = 0.2$ or $P(1|X = 0) = 0.6$.
  - $\beta_1 = \log(1.1)$ (L) or $\beta_1 = \log(2)$ (H).
  - 10% (L) or 50% (H) missing data in X_1.

  This led to eight scenarios ($S1, \ldots, S8$):

  1. $\beta_0 = -1.39$, $\beta_1 = \log(1.1)$, 10% missing (LLL).
  2. $\beta_0 = 0.405$, $\beta_1 = \log(1.1)$, 10% missing (HLL).
  3. $\beta_0 = -1.39$, $\beta_1 = \log(2)$, 10% missing (LHL).
  4. $\beta_0 = 0.405$, $\beta_1 = \log(2)$, 10% missing (HHL).
  5. $\beta_0 = -1.39$, $\beta_1 = \log(1.1)$, 50% missing (LLH).
  6. $\beta_0 = 0.405$, $\beta_1 = \log(1.1)$, 50% missing (HLH).
  7. $\beta_0 = -1.39$, $\beta_1 = \log(2)$, 50% missing (LHH).
  8. $\beta_0 = 0.405$, $\beta_1 = \log(2)$, 50% missing (HHH).

  The parameters $\beta_2, \beta_3, \beta_4$ were kept fixed at $\log(2), \log(0.85), \log(0.75)$.
- Cross-validation was then applied as explained in the main paper, with either $K = 1$, $K = 10$ or $K = 100$ imputations. For all analyses, ten folds were used ($L = 10$). Logistic regression models with predictors $X_1, \ldots, X_4$ were fitted.
- All analyses were replicated ten times.
- As measures of performance, we considered variation - as defined in the main paper - ('var'), bias (as the difference between the 'true' predicted probabilities as given by the generating model and the predicted probabilities in the validation folds after applying the whole procedure of MI and CV) and Brier score ('BS'). After analyzing the full datasets, all measures were assessed

on individuals with (M) and without (FO) (Fully Observed) missing values separately. We also add the Monte Carlo Standard Errors (MC SE), defined by $\sqrt{\frac{1}{(n_{sim})(n_{sim}-1)}\Sigma_{i=1}^{n_{sim}}(\hat{\theta}_i - \bar{\theta})^2}$, where $n_{sim} = 100$, $\hat{\theta}_i$ is the estimand of interest (variance, bias or Brier score) in the ith simulated dataset (averaged over the replications) and $\bar{\theta}$ the estimand averaged over the datasets. All statistics shown in the tables have been multiplied by a factor 100 to improve readability.

- The first two approaches introduced in the paper were implemented (A1 and A2).

1.2 R code

The files needed to repeat the simulation study are available as supplementary material. The code is structured in such a way that 100 datasets can be generated and analyzed in parallel by making use of a computer cluster. The files can be used as follows:

- 'one_simulation_logreg.R' sets the parameters for generating and analyzing one dataset for each of the eight scenarios. MCAR or MAR and the number of imputations have to be set manually. Data are generated and analyzed in the function 'sim_scenario'.
- 'sim_scenario' is found in 'sim_scenario_MCAR_logreg.R' or 'sim_scenario_MAR_logreg.R'. After the generation of the dataset, data are set to missing according to the chosen mechanism. It is analyzed 10 times according to the two approaches (A1, A2). The output contains both the generated dataset and the cross-validated predictions under the different approaches for different replications.
- The tables below have been created by summarizing over replications and simulations. The code is given in 'SummaryMeasures_function_logreg_ext.R' and 'SummaryMeasures_FinalTable_logreg_MCAR_M10.R' (again manually setting MCAR or MAR and the number of imputations).

1.3 Results

1.3.1 Tables

Listing of algorithms and tables presented

1. Algorithmic description of approach 1 (page 5, compatible to graphical representation in figure 1 of the paper)
2. Algorithmic description of approaches 2 and 3 (page 6, compatible to graphical representation in figure 2 of the paper)
3. Variance, bias and Brier statistics for MCAR simulation with $K = 1$ imputations (table S2)
4. Variance, bias and Brier statistics for MCAR simulation with $K = 10$ imputations (table S3)
5. Variance, bias and Brier statistics for MCAR simulation with $K = 100$ imputations (table S4)
6. Variance, bias and Brier statistics for MAR simulation with $K = 1$ imputations (table S5)
7. Variance, bias and Brier statistics for MAR simulation with $K = 10$ imputations (table S6)
8. Variance, bias and Brier statistics for MAR simulation with $K = 100$ imputations (table S7)

We repeat the scenarios table (table S1) from the paper for ease of reference.

Approach 1

Define K and repeat the following steps K times

Define L folds for the CV procedure. Select each fold in turn as the validation data, keeping all other data as calibration set. For each such fold carry out the following computation.

1. Remove the outcome from the validation data.
2. Combine this outcome-deleted validation data with the corresponding calibration data. Generate a single imputation on this combined set.
3. Fit a logistic regression model in the imputations-augmented calibration portion of this combined set.
4. Derive predictions for subjects i in the corresponding imputed validation set from this model, using the equation

$$\widehat{P}_{i,k} = exp\{\widehat{\boldsymbol{x}}_{i,k}^T \widehat{\boldsymbol{\beta}}_k\}/(1 + exp\{\widehat{\boldsymbol{x}}_{i,k}^T \widehat{\boldsymbol{\beta}}_k\}).$$

Compute the final prediction for each ith individual as the average $\overline{P}_i$ across all K predictions $\widehat{P}_{i,k}$.

Algorithm 1. Algorithmic description of approach 1 for combination of multiple imputation with cross-validation using the logistic regression modelling for binary outcome. K represents the number of imputations and L denotes the number of folds in the cross-validation. Note that the folds get re-defined for each new imputation in this approach. $\widehat{\boldsymbol{\beta}}_k$ denotes the vector of regression coefficients fitted in the calibration fold for the kth imputation. The notation $\widehat{\boldsymbol{x}}_{i,k}$ denotes the imputed data record for the set-aside i^{th} patient in the validation fold for the kth imputation (observed data are not affected).

Approaches 2 and 3

Define L folds for the CV procedure and select each lth fold in turn as the validation set, keeping all other data as calibration set. For each such fold carry out the following computation.

1. Remove the outcome from the validation data.
2. Combine this outcome-deleted validation data with the corresponding calibration data.
3. Run K imputations on this combined set.
4. Fit separate logistic regression models on the training portion of each of these K imputed datasets.
5. Compute the average $\bar{\boldsymbol{\beta}}$ of the K regression coefficients vectors from these models.
6. Derive predictions for subjects i in the imputed validation sets from the combined model, using the equation

$$\widehat{P}_{i,k} = exp\{\widehat{\boldsymbol{x}}_{i,k}^T \bar{\boldsymbol{\beta}}\}/(1 + exp\{\widehat{\boldsymbol{x}}_{i,k}^T \bar{\boldsymbol{\beta}}\})$$

Compute the final prediction for each ith individual as the average $\overline{P}_i$ across all K predictions $\widehat{P}_{i,k}$.

Algorithm 2. Algorithmic description of approach 2 for combination of multiple imputation with cross-validation using the logistic regression modelling for binary outcome. K represents the number of imputations and L denotes the number of folds in the cross-validation. Note that the folds get defined first in this approach and are then held fixed. $\bar{\boldsymbol{\beta}}$ denotes the Rubin's-rule pooled regression coefficients across imputations fitted in the calibration fold. $\widehat{\boldsymbol{x}}_{i,k}$ denotes the imputed data record for the set-aside i^{th} patient in the validation fold for the kth imputation. Approach 3 is identical to approach 2, except that the individual imputations $\widehat{\boldsymbol{x}}_{i,k}$ are replaced with the Rubin's rule pooled imputation $\bar{\boldsymbol{x}}_i$, such that only a single prediction $\widehat{P}_i$ is generated.

scenario	β_0	β_1	% missing	shorthand reference notation
1	-1.39	log(1.1)	10%	LLL
2	0.405	log(1.1)	10%	HLL
3	-1.39	log(2)	10%	LHL
4	0.405	log(2)	10%	HHL
5	-1.39	log(1.1)	50%	LLH
6	0.405	log(1.1)	50%	HLH
7	-1.39	log(2)	50%	LHH
8	0.405	log(2)	50%	HHH

Table S1: Summary description of the simulation scenarios investigated. A 2^3 design is used, corresponding to all combinations of either Low (L) or High (H) for the parameters β_0, β_1 and the percentage of missing values. These scenarios are investigated in both the MCAR and MAR cases.

Scenario - MCAR			Method	Variance $\overline{R}$ (MC SE)				Bias $\overline{\overline{Bias}}$ (MC SE)				Brier score $\overline{\overline{B}}$ (MC SE)			
β_0	β_1	M		Missing data		Fully observed		Missing data		Fully observed		Missing data		Fully observed	
$K = 1$															
L	L	L	A1	5.76	(0.26)	3.01	(0.01)	-0.03	(0.18)	0.22	(0.18)	16.14	(0.22)	15.99	(0.07)
L	L	L	A2	5.86	(0.27)	3.01	(0.01)	-0.03	(0.18)	0.22	(0.18)	16.14	(0.22)	15.99	(0.07)
H	L	L	A1	5.67	(0.25)	2.86	(0.01)	0.21	(0.15)	0.15	(0.16)	22.22	(0.16)	22.19	(0.06)
H	L	L	A2	5.72	(0.25)	2.87	(0.01)	0.20	(0.16)	0.15	(0.16)	22.23	(0.16)	22.19	(0.06)
L	H	L	A1	23.07	(0.42)	2.98	(0.02)	-6.07	(0.18)	-0.14	(0.21)	17.83	(0.24)	16.02	(0.07)
L	H	L	A2	23.11	(0.41)	3.00	(0.02)	-6.08	(0.18)	-0.14	(0.21)	17.80	(0.24)	16.02	(0.07)
H	H	L	A1	31.57	(0.36)	2.93	(0.01)	1.10	(0.20)	-0.07	(0.15)	25.47	(0.12)	22.17	(0.05)
H	H	L	A2	31.60	(0.37)	2.93	(0.01)	1.06	(0.20)	-0.07	(0.15)	25.47	(0.13)	22.17	(0.05)
L	L	H	A1	7.61	(0.31)	4.50	(0.03)	-0.03	(0.20)	0.09	(0.20)	16.02	(0.10)	16.02	(0.11)
L	L	H	A2	7.65	(0.32)	4.53	(0.03)	-0.04	(0.20)	0.10	(0.20)	16.01	(0.10)	16.02	(0.11)
H	L	H	A1	7.15	(0.30)	4.33	(0.03)	0.03	(0.15)	0.02	(0.15)	22.32	(0.06)	22.33	(0.07)
H	L	H	A2	7.15	(0.30)	4.33	(0.02)	0.03	(0.15)	0.03	(0.15)	22.32	(0.06)	22.33	(0.07)
L	H	H	A1	23.84	(0.46)	4.63	(0.04)	-6.41	(0.15)	-0.03	(0.22)	17.94	(0.11)	15.90	(0.10)
L	H	H	A2	23.84	(0.44)	4.57	(0.04)	-6.38	(0.15)	-0.04	(0.22)	17.92	(0.11)	15.90	(0.10)
H	H	H	A1	30.61	(0.48)	4.61	(0.03)	1.07	(0.15)	-0.22	(0.15)	25.35	(0.07)	22.35	(0.06)
H	H	H	A2	30.62	(0.49)	4.63	(0.03)	1.03	(0.15)	-0.21	(0.15)	25.36	(0.07)	22.35	(0.06)

Table S2: MCAR simulations with $K = 1$. All statistics were multiplied by 100. L=low, H=high. A1 and A2 denotes approaches 1 or 2 respectively.

Scenario - MCAR			Method	Variance $\overline{R}$ (MC SE)				Bias $\overline{\overline{Bias}}$ (MC SE)				Brier score $\overline{\overline{B}}$ (MC SE)			
β_0	β_1	M		Missing data		Fully observed		Missing data		Fully observed		Missing data		Fully observed	
$K = 10$															
L	L	L	A1	1.87	(0.08)	0.95	(0.00)	-0.04	(0.18)	0.23	(0.18)	16.1	(0.22)	16.0	(0.07)
L	L	L	A2	2.99	(0.06)	2.75	(0.01)	-0.05	(0.18)	0.22	(0.18)	16.1	(0.22)	16.0	(0.07)
H	L	L	A1	1.87	(0.10)	0.91	(0.00)	-0.35	(0.17)	-0.36	(0.17)	22.4	(0.15)	22.3	(0.05)
H	L	L	A2	2.94	(0.07)	2.66	(0.01)	-0.35	(0.17)	-0.36	(0.17)	22.4	(0.15)	22.3	(0.05)
L	H	L	A1	7.45	(0.12)	0.95	(0.00)	-6.15	(0.21)	-0.08	(0.21)	17.0	(0.23)	15.9	(0.08)
L	H	L	A2	7.80	(0.11)	2.75	(0.01)	-6.12	(0.21)	-0.09	(0.21)	17.0	(0.23)	15.9	(0.08)
H	H	L	A1	9.95	(0.13)	0.93	(0.00)	1.22	(0.19)	-0.01	(0.17)	23.9	(0.12)	22.1	(0.05)
H	H	L	A2	10.15	(0.12)	2.67	(0.01)	1.22	(0.19)	-0.01	(0.17)	23.9	(0.12)	22.1	(0.05)
L	L	H	A1	2.37	(0.08)	1.43	(0.01)	0.16	(0.20)	0.25	(0.20)	16.2	(0.11)	16.1	(0.11)
L	L	H	A2	3.09	(0.07)	3.15	(0.02)	0.14	(0.20)	0.22	(0.20)	16.2	(0.11)	16.1	(0.11)
H	L	H	A1	2.38	(0.09)	1.36	(0.00)	-0.25	(0.16)	-0.27	(0.16)	22.2	(0.07)	22.2	(0.06)
H	L	H	A2	3.10	(0.07)	3.08	(0.01)	-0.24	(0.16)	-0.27	(0.16)	22.2	(0.07)	22.2	(0.06)
L	H	H	A1	7.52	(0.15)	1.45	(0.01)	-6.54	(0.14)	-0.20	(0.21)	17.0	(0.10)	15.9	(0.10)
L	H	H	A2	7.74	(0.15)	3.13	(0.02)	-6.57	(0.14)	-0.22	(0.21)	17.0	(0.10)	15.9	(0.10)
H	H	H	A1	10.09	(0.16)	1.46	(0.01)	1.23	(0.13)	0.12	(0.14)	23.9	(0.05)	22.2	(0.06)
H	H	H	A2	10.33	(0.16)	3.09	(0.01)	1.23	(0.13)	0.13	(0.14)	23.9	(0.05)	22.2	(0.06)

Table S3: MCAR simulations with $K = 10$. All statistics were multiplied by 100. L=low, H=high. A1 and A2 denotes approaches 1 or 2 respectively.

Scenario - MCAR			Method	Variance $\overline{R}$ (MC SE)				Bias $\overline{\overline{Bias}}$ (MC SE)				Brier score $\overline{\overline{B}}$ (MC SE)			
β_0	β_1	M		Missing data		Fully observed		Missing data		Fully observed		Missing data		Fully observed	
$K = 100$															
L	L	L	A1	0.59	(0.03)	0.30	(0.00)	-0.04	(0.18)	0.23	(0.18)	16.1	(0.22)	16.0	(0.07)
L	L	L	A2	2.41	(0.02)	2.72	(0.01)	-0.04	(0.18)	0.22	(0.18)	16.1	(0.22)	16.0	(0.07)
H	L	L	A1	0.57	(0.03)	0.29	(0.00)	-0.20	(0.15)	-0.16	(0.14)	22.1	(0.16)	22.3	(0.05)
H	L	L	A2	2.40	(0.02)	2.65	(0.01)	-0.20	(0.15)	-0.16	(0.14)	22.1	(0.16)	22.3	(0.05)
L	H	L	A1	2.32	(0.04)	0.30	(0.00)	-6.32	(0.22)	-0.25	(0.21)	16.9	(0.24)	16.0	(0.08)
L	H	L	A2	3.15	(0.03)	2.72	(0.01)	-6.32	(0.22)	-0.25	(0.21)	16.9	(0.24)	16.0	(0.08)
H	H	L	A1	3.08	(0.04)	0.29	(0.00)	1.35	(0.17)	0.16	(0.13)	23.9	(0.13)	22.1	(0.05)
H	H	L	A2	3.93	(0.03)	2.64	(0.01)	1.34	(0.17)	0.16	(0.13)	23.9	(0.13)	22.1	(0.05)
L	L	H	A1	0.77	(0.03)	0.45	(0.00)	-0.40	(0.16)	-0.28	(0.17)	16.0	(0.11)	16.0	(0.10)
L	L	H	A2	2.47	(0.02)	2.93	(0.02)	-0.43	(0.16)	-0.32	(0.17)	16.0	(0.11)	16.0	(0.10)
H	L	H	A1	0.80	(0.03)	0.43	(0.00)	-0.12	(0.16)	-0.13	(0.16)	22.3	(0.07)	22.1	(0.07)
H	L	H	A2	2.42	(0.02)	2.85	(0.01)	-0.11	(0.16)	-0.12	(0.16)	22.3	(0.07)	22.2	(0.07)
L	H	H	A1	2.50	(0.04)	0.46	(0.00)	-6.17	(0.16)	0.29	(0.18)	16.9	(0.10)	16.0	(0.10)
L	H	H	A2	3.35	(0.03)	3.01	(0.02)	-6.19	(0.16)	0.28	(0.18)	16.9	(0.10)	16.0	(0.10)
H	H	H	A1	3.04	(0.05)	0.46	(0.00)	1.43	(0.15)	0.31	(0.18)	23.8	(0.05)	22.2	(0.07)
H	H	H	A2	3.92	(0.04)	2.89	(0.01)	1.44	(0.15)	0.31	(0.18)	23.8	(0.05)	22.2	(0.07)

Table S4: MCAR simulations with $K = 100$. All statistics were multiplied by 100. L=low, H=high. A1 and A2 denotes approaches 1 or 2 respectively.

Scenario - MAR			Method	Variance $\overline{R}$ (MC SE)				Bias $\overline{\overline{Bias}}$ (MC SE)				Brier score $\overline{\overline{B}}$ (MC SE)			
β_0	β_1	M		Missing data		Fully observed		Missing data		Fully observed		Missing data		Fully observed	
$K = 1$															
L	L	L	A1	6.01	(0.28)	3.04	(0.01)	0.14	(0.19)	0.21	(0.17)	17.33	(0.23)	15.86	(0.07)
L	L	L	A2	6.00	(0.29)	3.03	(0.02)	0.13	(0.19)	0.21	(0.17)	17.34	(0.23)	15.86	(0.07)
H	L	L	A1	5.67	(0.27)	2.89	(0.01)	-0.10	(0.15)	0.01	(0.14)	21.95	(0.18)	22.37	(0.05)
H	L	L	A2	5.67	(0.26)	2.90	(0.01)	-0.10	(0.15)	0.00	(0.14)	21.94	(0.18)	22.38	(0.05)
L	H	L	A1	24.52	(0.39)	3.03	(0.01)	-6.13	(0.19)	-0.04	(0.19)	18.35	(0.24)	15.86	(0.07)
L	H	L	A2	24.65	(0.39)	3.03	(0.02)	-6.07	(0.18)	-0.05	(0.19)	18.32	(0.22)	15.86	(0.07)
H	H	L	A1	31.43	(0.42)	2.92	(0.01)	1.47	(0.21)	0.09	(0.16)	24.95	(0.15)	22.12	(0.05)
H	H	L	A2	31.52	(0.42)	2.91	(0.01)	1.40	(0.21)	0.08	(0.16)	24.98	(0.14)	22.12	(0.05)
L	L	H	A1	7.49	(0.28)	4.55	(0.04)	0.04	(0.21)	-0.07	(0.18)	17.55	(0.12)	14.46	(0.11)
L	L	H	A2	7.51	(0.27)	4.61	(0.04)	0.05	(0.21)	-0.08	(0.18)	17.56	(0.12)	14.46	(0.11)
H	L	H	A1	7.62	(0.33)	4.27	(0.03)	-0.00	(0.17)	-0.01	(0.16)	21.67	(0.09)	22.87	(0.06)
H	L	H	A2	7.62	(0.33)	4.29	(0.03)	-0.00	(0.17)	-0.01	(0.16)	21.67	(0.09)	22.88	(0.06)
L	H	H	A1	24.86	(0.50)	4.64	(0.04)	-5.57	(0.17)	0.33	(0.23)	18.83	(0.12)	15.48	(0.10)
L	H	H	A2	24.97	(0.49)	4.69	(0.04)	-5.57	(0.16)	0.34	(0.23)	18.82	(0.12)	15.48	(0.10)
H	H	H	A1	31.06	(0.54)	4.60	(0.03)	1.67	(0.16)	0.18	(0.17)	25.08	(0.08)	22.38	(0.07)
H	H	H	A2	31.15	(0.56)	4.62	(0.03)	1.65	(0.16(	0.17	(0.17)	25.09	(0.09)	22.38	(0.07)

Table S5: MAR simulations with $K = 1$. All statistics were multiplied by 100. L=low, H=high. A1 and A2 denotes approaches 1 or 2 respectively.

Scenario - MAR			Method	Variance $\overline{R}$ (MC SE)				Bias $\overline{\overline{Bias}}$ (MC SE)				Brier score $\overline{\overline{B}}$ (MC SE)			
β_0	β_1	M		Missing data		Fully observed		Missing data		Fully observed		Missing data		Fully observed	
$K = 10$															
L	L	L	A1	1.95	(0.09)	0.96	(0.00)	0.13	(0.20)	0.21	(0.17)	17.3	(0.23)	15.8	(0.07)
L	L	L	A2	3.10	(0.06)	2.76	(0.01)	0.13	(0.20)	0.20	(0.17)	17.3	(0.23)	15.9	(0.07)
H	L	L	A1	1.66	(0.07)	0.91	(0.00)	0.11	(0.14)	0.06	(0.13)	21.7	(0.16)	22.4	(0.05)
H	L	L	A2	2.73	(0.05)	2.67	(0.01)	0.11	(0.14)	0.06	(0.13)	21.7	(0.16)	22.4	(0.05)
L	H	L	A1	7.83	(0.11)	0.96	(0.00)	-5.89	(0.19)	0.03	(0.17)	17.7	(0.23)	16.0	(0.08)
L	H	L	A2	8.04	(0.10)	2.77	(0.01)	-5.91	(0.19)	0.03	(0.17)	17.7	(0.23)	16.0	(0.08)
H	H	L	A1	9.52	(0.12)	0.92	(0.00)	1.10	(0.23)	-0.10	(0.18)	23.5	(0.12)	22.2	(0.05)
H	H	L	A2	9.82	(0.11)	2.67	(0.01)	1.14	(0.22)	-0.10	(0.18)	23.5	(0.12)	22.2	(0.05)
L	L	H	A1	2.53	(0.09)	1.46	(0.01)	0.02	(0.22)	0.18	(0.19)	17.5	(0.10)	14.6	(0.10)
L	L	H	A2	3.26	(0.08)	3.12	(0.02)	-0.00	(0.22)	0.15	(0.19)	17.5	(0.10)	14.6	(0.10)
H	L	H	A1	2.32	(0.09)	1.36	(0.01)	0.06	(0.15)	0.01	(0.16)	21.7	(0.07)	22.9	(0.06)
H	L	H	A2	3.02	(0.07)	3.07	(0.01)	0.07	(0.15)	0.02	(0.16)	21.7	(0.07)	22.9	(0.06)
L	H	H	A1	7.48	(0.14)	1.46	(0.01)	-5.95	(0.17)	-0.16	(0.19)	17.7	(0.11)	15.5	(0.10)
L	H	H	A2	7.73	(0.14)	3.13	(0.02)	-5.96	(0.17)	-0.18	(0.19)	17.7	(0.11)	15.5	(0.10)
H	H	H	A1	9.78	(0.16)	1.46	(0.01)	1.40	(0.15)	-0.19	(0.16)	23.6	(0.06)	22.3	(0.07)
H	H	H	A2	10.04	(0.16)	3.10	(0.01)	1.41	(0.15)	-0.18	(0.16)	23.6	(0.06)	22.3	(0.07)

Table S6: MAR simulations with $K = 10$. All statistics were multiplied by 100. L=low, H=high. A1 and A2 denotes approaches 1 or 2 respectively.

Scenario - MAR			Method	Variance $\overline{R}$ (MC SE)				Bias $\overline{\overline{Bias}}$ (MC SE)				Brier score $\overline{\overline{B}}$ (MC SE)			
β_0	β_1	M		Missing data		Fully observed		Missing data		Fully observed		Missing data		Fully observed	
$K = 100$															
L	L	L	A1	0.62	(0.03)	0.30	(0.00)	0.13	(0.20)	0.21	(0.17)	17.3	(0.23)	15.8	(0.07)
L	L	L	A2	2.56	(0.02)	2.73	(0.01)	0.14	(0.20)	0.20	(0.17)	17.3	(0.23)	15.9	(0.07)
H	L	L	A1	0.52	(0.02)	0.29	(0.00)	0.11	(0.17)	-0.03	(0.16)	21.6	(0.18)	22.3	(0.05)
H	L	L	A2	2.29	(0.01)	2.63	(0.01)	0.12	(0.17)	-0.03	(0.16)	21.6	(0.18)	22.3	(0.05)
L	H	L	A1	2.46	(0.04)	0.30	(0.00)	-5.75	(0.20)	0.01	(0.20)	17.9	(0.24)	15.9	(0.07)
L	H	L	A2	3.33	(0.03)	2.75	(0.01)	-5.76	(0.20)	0.01	(0.20)	17.9	(0.25)	15.9	(0.07)
H	H	L	A1	3.05	(0.04)	0.29	(0.00)	1.48	(0.19)	-0.01	(0.15)	23.6	(0.14)	22.2	(0.06)
H	H	L	A2	3.88	(0.04)	2.63	(0.01)	1.48	(0.19)	-0.01	(0.15)	23.6	(0.14)	22.2	(0.06)
L	L	H	A1	0.91	(0.04)	0.46	(0.00)	-0.07	(0.21)	0.08	(0.20)	17.5	(0.10)	14.4	(0.11)
L	L	H	A2	2.65	(0.02)	2.88	(0.02)	-0.10	(0.21)	0.06	(0.20)	17.5	(0.10)	14.4	(0.11)
H	L	H	A1	0.73	(0.03)	0.43	(0.00)	0.09	(0.16)	0.10	(0.15)	21.6	(0.07)	22.8	(0.06)
H	L	H	A2	2.34	(0.02)	2.89	(0.01)	0.10	(0.16)	0.10	(0.15)	21.6	(0.07)	22.8	(0.06)
L	H	H	A1	2.46	(0.05)	0.46	(0.00)	-6.05	(0.15)	-0.14	(0.22)	17.5	(0.09)	15.3	(0.09)
L	H	H	A2	3.37	(0.04)	2.92	(0.02)	-6.08	(0.15)	-0.15	(0.22)	17.6	(0.09)	15.3	(0.09)
H	H	H	A1	3.11	(0.05)	0.46	(0.00)	1.54	(0.18)	0.18	(0.18)	23.5	(0.06)	22.4	(0.07)
H	H	H	A2	3.97	(0.04)	2.93	(0.01)	1.54	(0.18)	0.18	(0.18)	23.5	(0.06)	22.4	(0.07)

Table S7: MAR simulations with $K = 100$. All statistics were multiplied by 100. L=low, H=high. A1 and A2 denotes approaches 1 or 2 respectively.

1.3.2 Figures

Listing of figures presented

1. Variance statistics for MCAR, scenarios 1-4
2. Variance statistics for MCAR, scenarios 5-8
3. Brier statistics for MCAR, scenarios 1-4
4. Brier statistics for MCAR, scenarios 5-8
5. Bias statistics for MCAR, scenarios 1-4
6. Bias statistics for MCAR, scenarios 5-8
7. Variance statistics for MAR, scenarios 1-4
8. Variance statistics for MAR, scenarios 5-8
9. Brier statistics for MAR, scenarios 1-4
10. Brier statistics for MAR, scenarios 5-8
11. Bias statistics for MAR, scenarios 1-4
12. Bias statistics for MAR, scenarios 5-8

Note that the plots showing variance measures in scenarios 1 (LLL) and 3 (LHL) for MCAR are repeated from the paper.

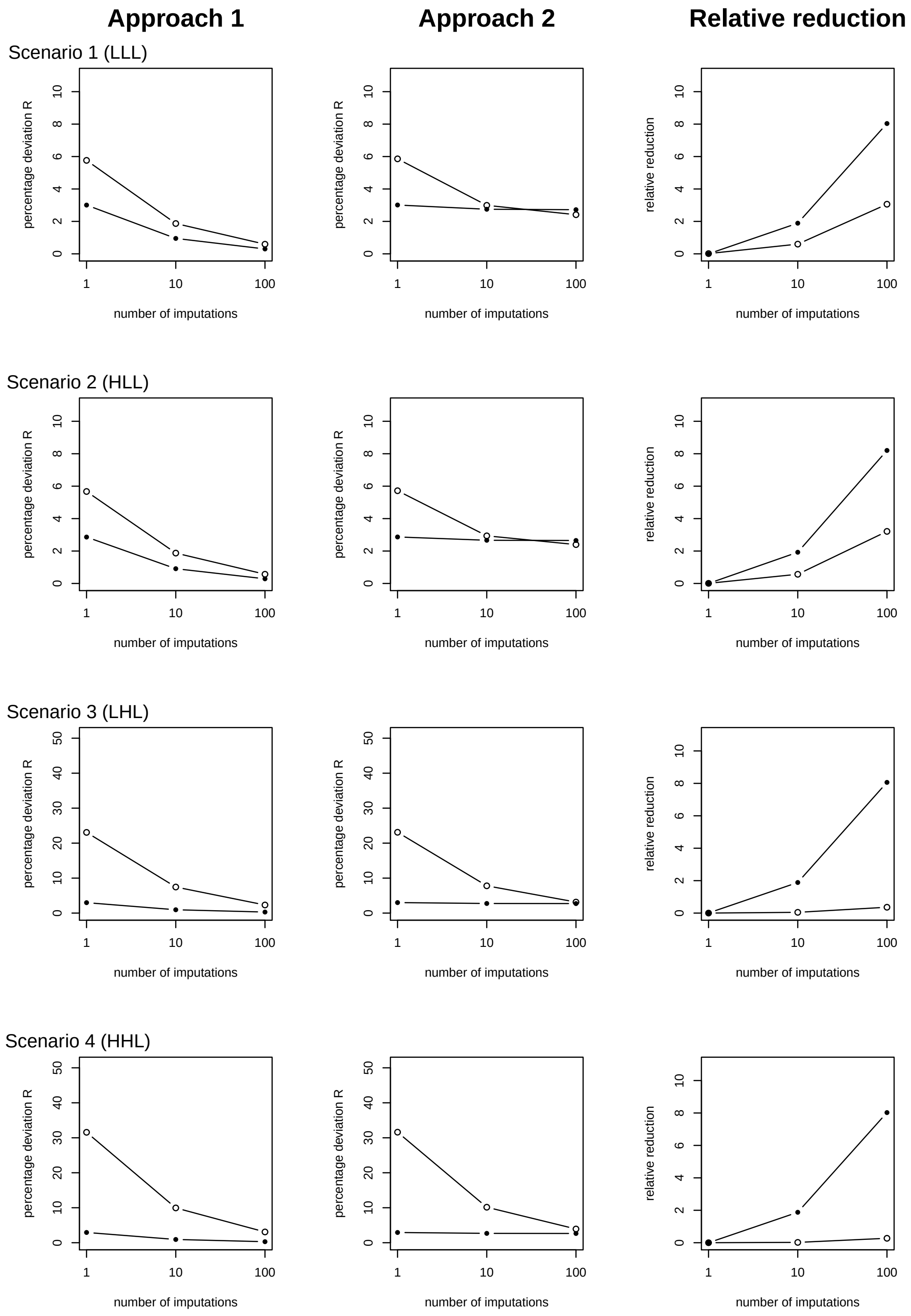

Figure S1: Average percentage prediction deviations measures ($\overline{R}$). The four rows of plots from top to bottom correspond to simulation scenarios 1 to 4, MCAR. The two left columns of plots show results from approaches 1 and 2 (as also shown in figures 4 and 6 of the paper) versus the number of imputations used in the calibration of the predictors. The right-side column of plots displays the corresponding relative variance reductions for approach 1 relative to approach 2. See table S1 for description of the scenarios.

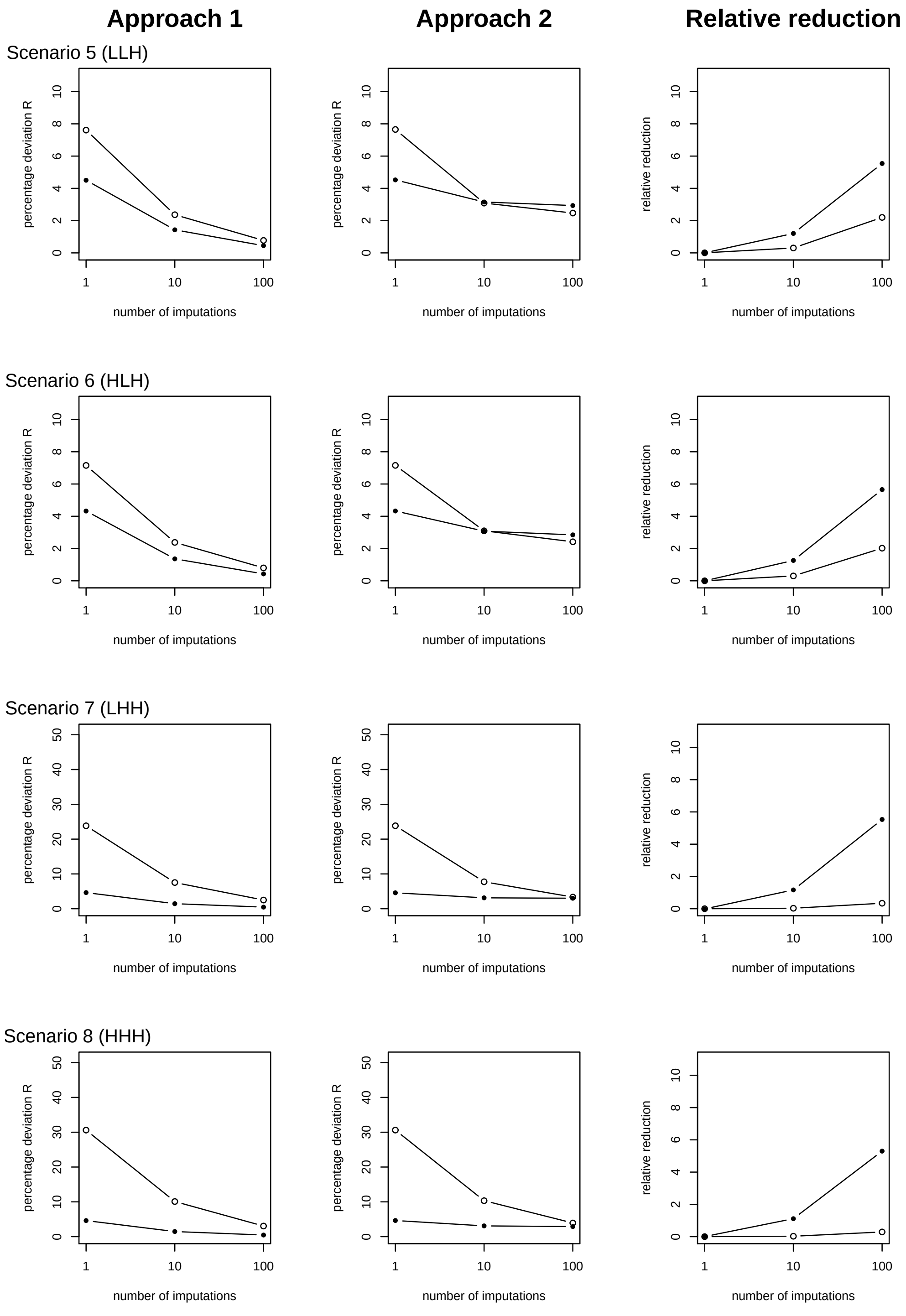

Figure S2: Average percentage prediction deviations measures ($\overline{R}$). The four rows of plots from top to bottom correspond to simulation scenarios 5 to 8, MCAR. The two left columns of plots show results from approaches 1 and 2 (as also shown in figures 4 and 6 of the paper) versus the number of imputations used in the calibration of the predictors. The right-side column of plots displays the corresponding relative variance reductions for approach 1 relative to approach 2. See table S1 for description of the scenarios.

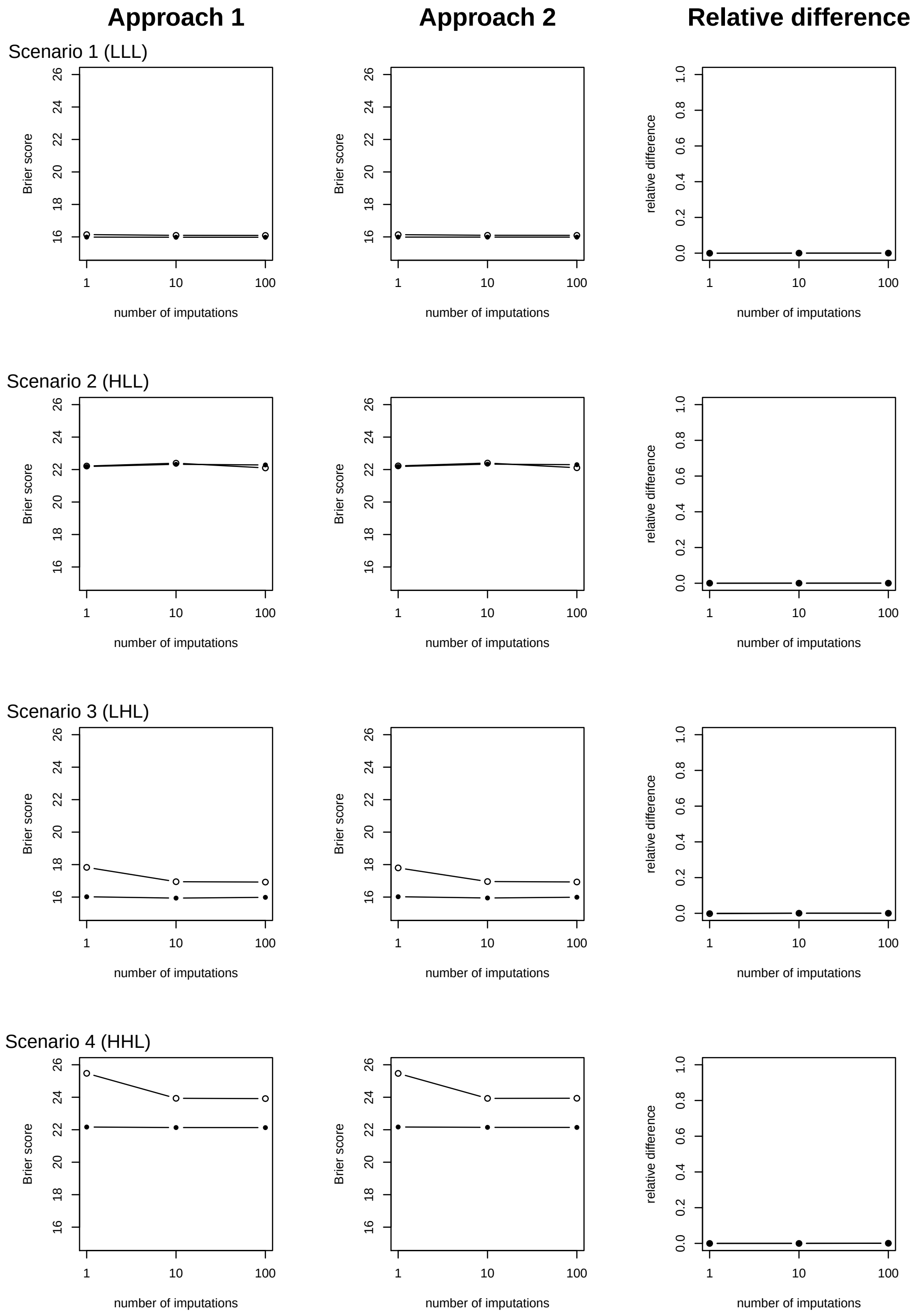

Figure S3: Average Brier scores ($\overline{\overline{B}}$). The four rows of plots from top to bottom correspond to simulation scenarios 1 to 4, MCAR (tables S2 to S4). The two left columns of plots show results from approaches 1 and 2 versus the number of imputations used in the calibration of the predictors. The right-side column of plots displays the corresponding relative reductions for approach 1 relative to approach 2. See table S1 for description of the scenarios.

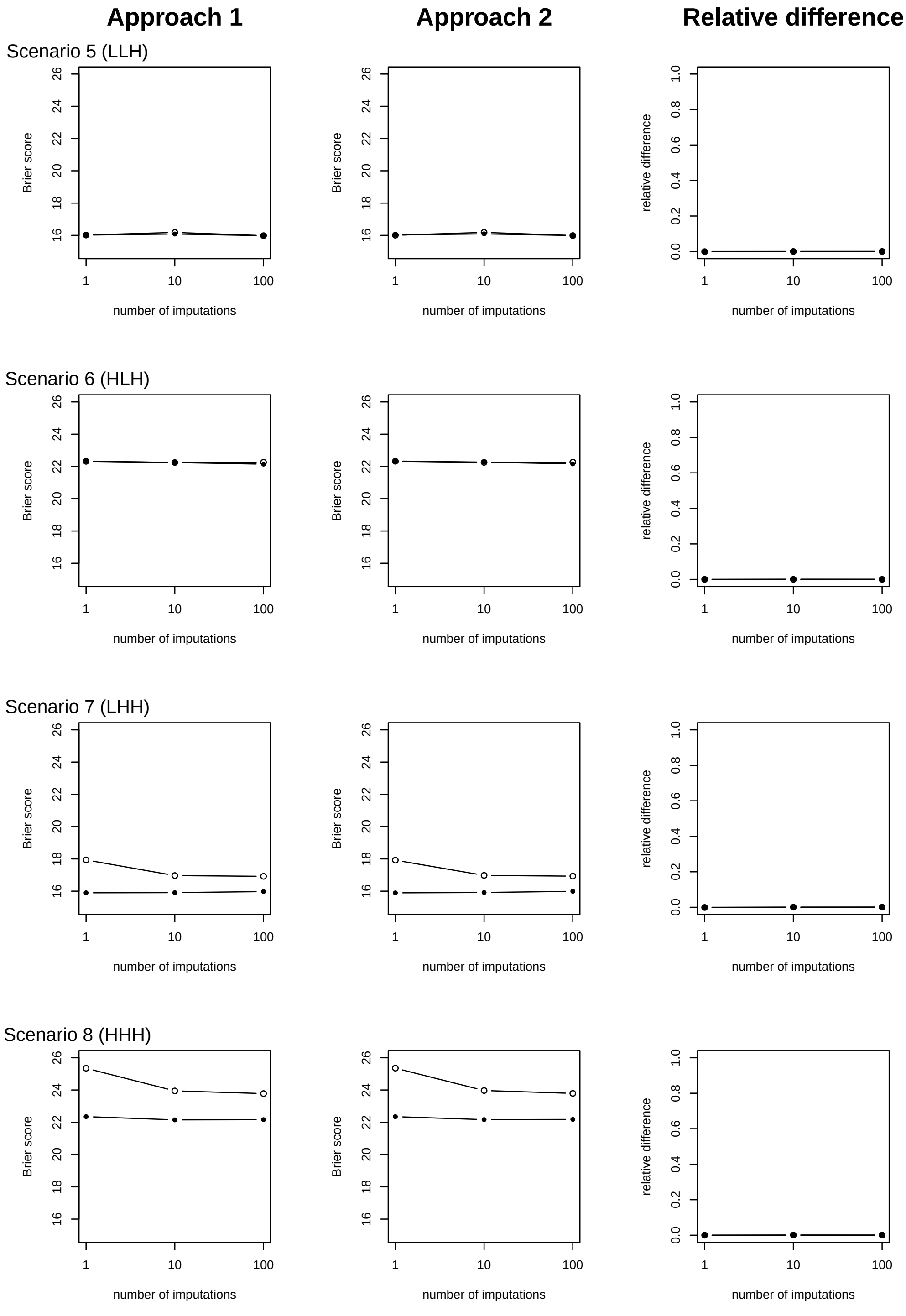

Figure S4: Average Brier scores ($\overline{\overline{B}}$). The four rows of plots from top to bottom correspond to simulation scenarios 5 to 8, MCAR (tables S2 to S4). The two left columns of plots show results from approaches 1 and 2 versus the number of imputations used in the calibration of the predictors. The right-side column of plots displays the corresponding relative reductions for approach 1 relative to approach 2. See table S1 for description of the scenarios.

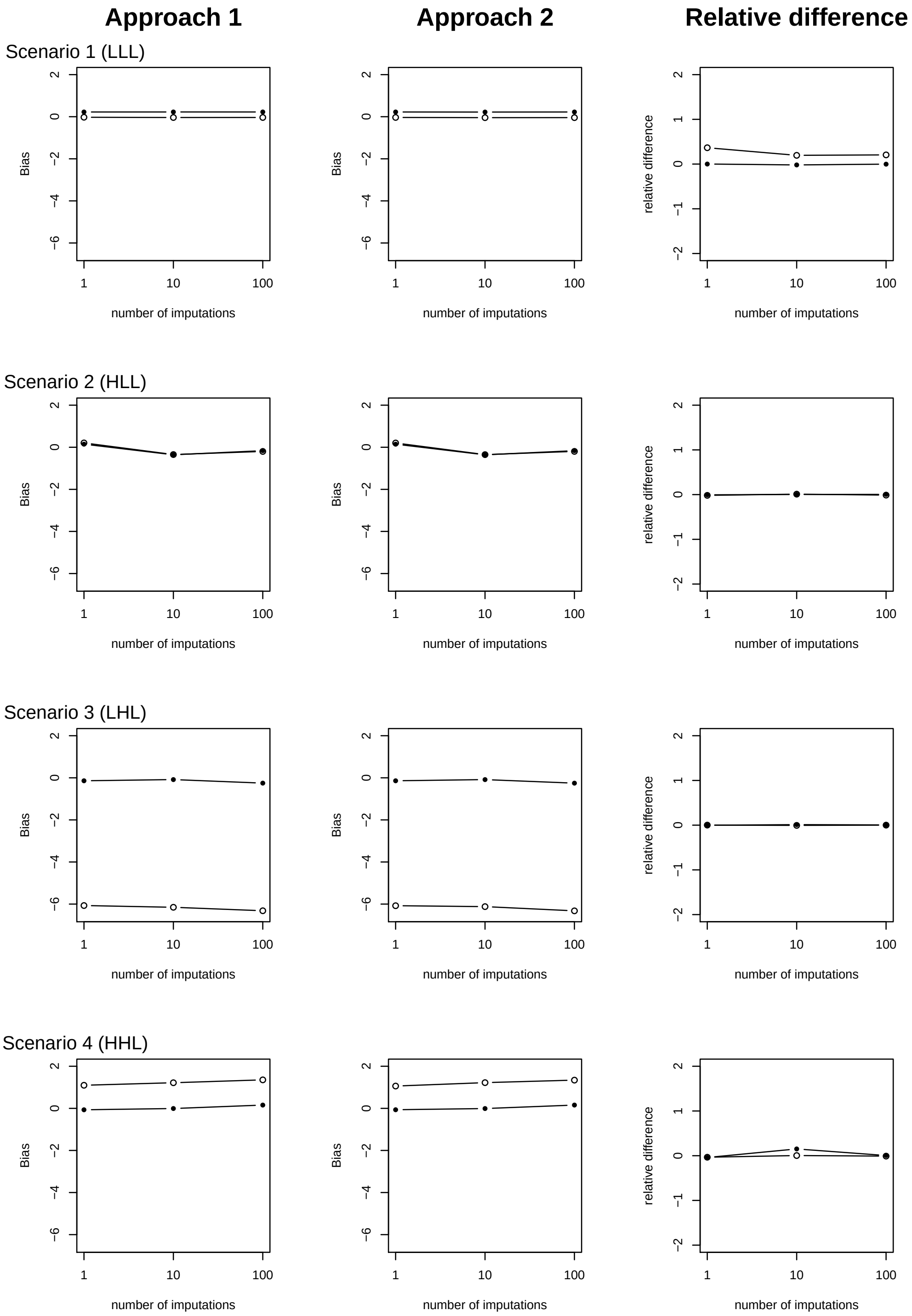

Figure S5: Average bias measures ($\overline{\overline{Bias}}$). The four rows of plots from top to bottom correspond to simulation scenarios 1 to 4, MCAR (tables S2 to S4). The two left columns of plots show results from approaches 1 and 2 versus the number of imputations used in the calibration of the predictors. The right-side column of plots displays the corresponding relative reductions for approach 1 relative to approach 2. See table S1 for description of the scenarios.

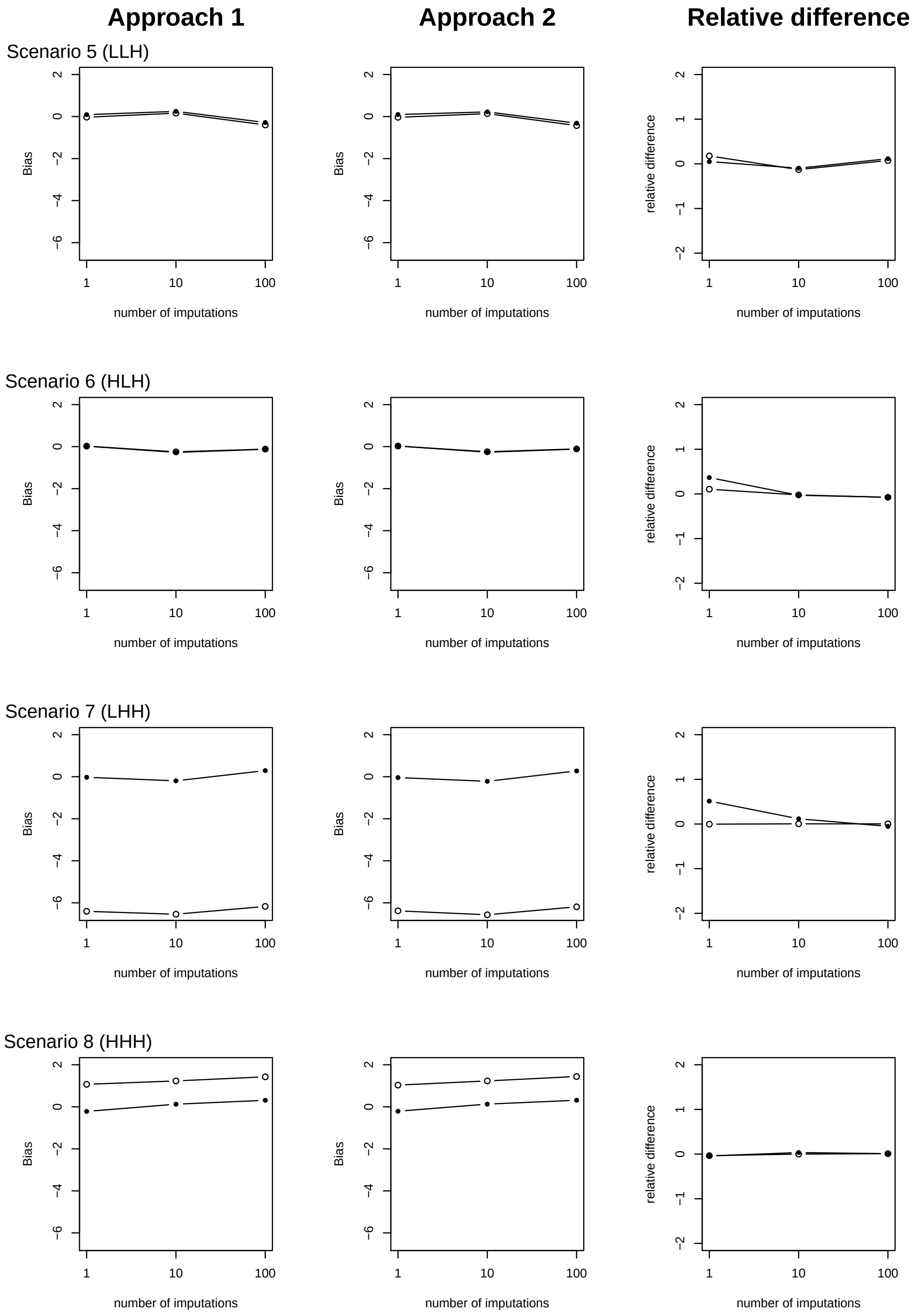

Figure S6: Average bias measures ($\overline{Bias}$). The four rows of plots from top to bottom correspond to simulation scenarios 5 to 8, MCAR (tables S2 to S4). The two left columns of plots show results from approaches 1 and 2 versus the number of imputations used in the calibration of the predictors. The right-side column of plots displays the corresponding relative reductions for approach 1 relative to approach 2. See table S1 for description of the scenarios.

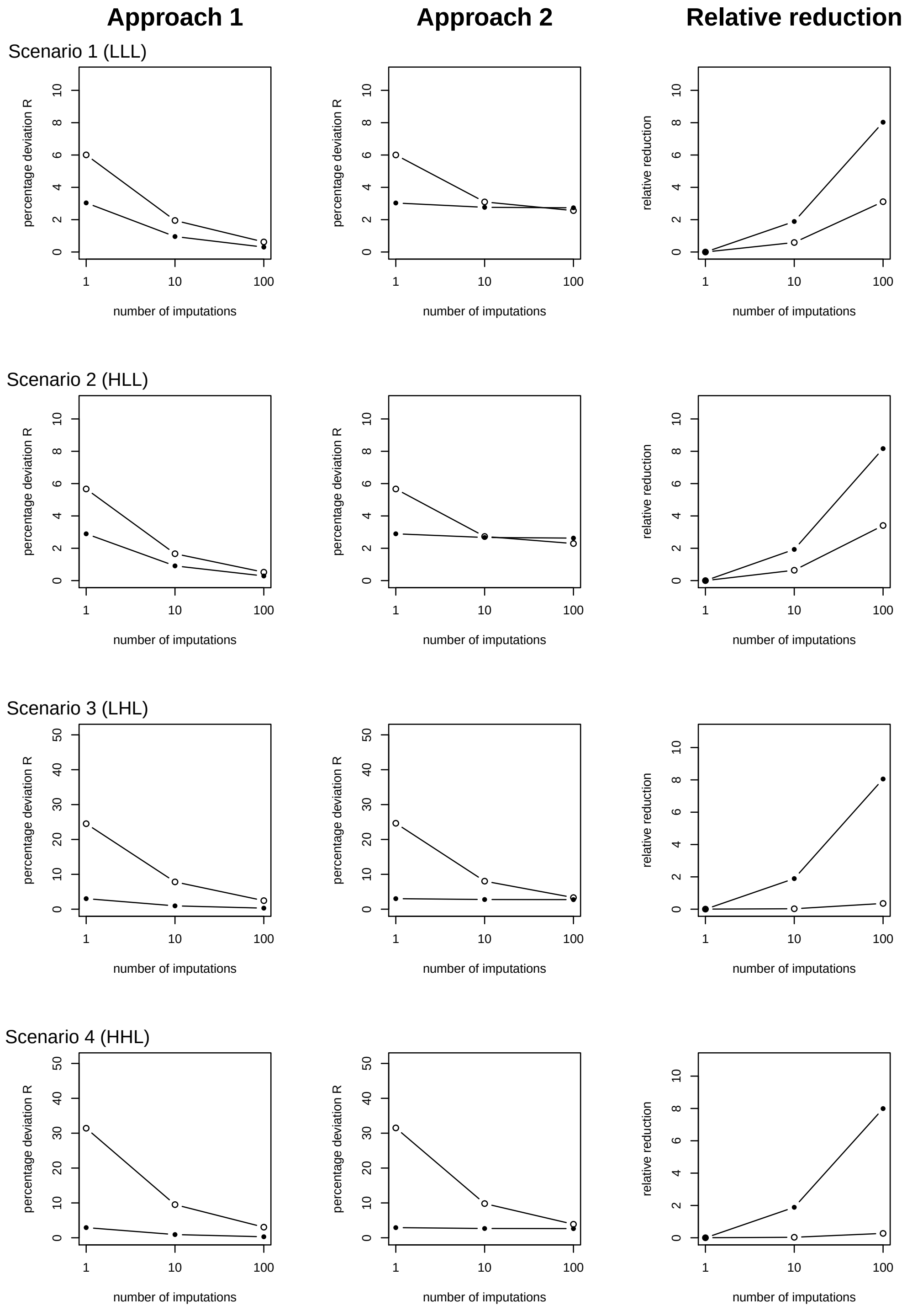

Figure S7: Average percentage prediction deviations measures ($\overline{R}$). The four rows of plots from top to bottom correspond to simulation scenarios 1 to 4, MAR (tables S5 to S7). The two left columns of plots show results from approaches 1 and 2 versus the number of imputations used in the calibration of the predictors. The right-side column of plots displays the corresponding relative variance reductions for approach 1 relative to approach 2. See table S1 for description of the scenarios.

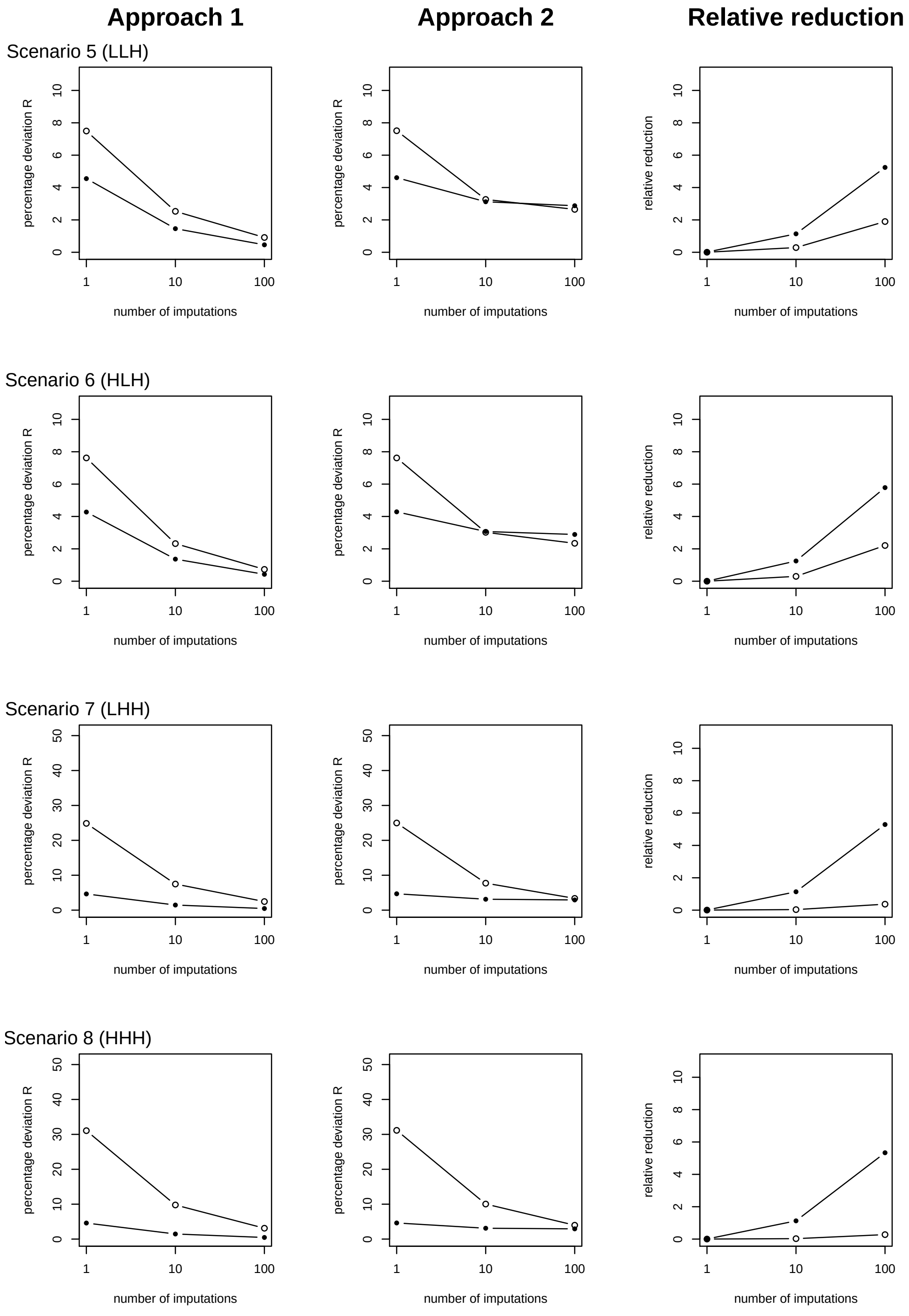

Figure S8: Average percentage prediction deviations measures ($\overline{R}$). The four rows of plots from top to bottom correspond to simulation scenarios 5 to 8, MAR (tables S5 to S7). The two left columns of plots show results from approaches 1 and 2 versus the number of imputations used in the calibration of the predictors. The right-side column of plots displays the corresponding relative variance reductions for approach 1 relative to approach 2. See table S1 for description of the scenarios.

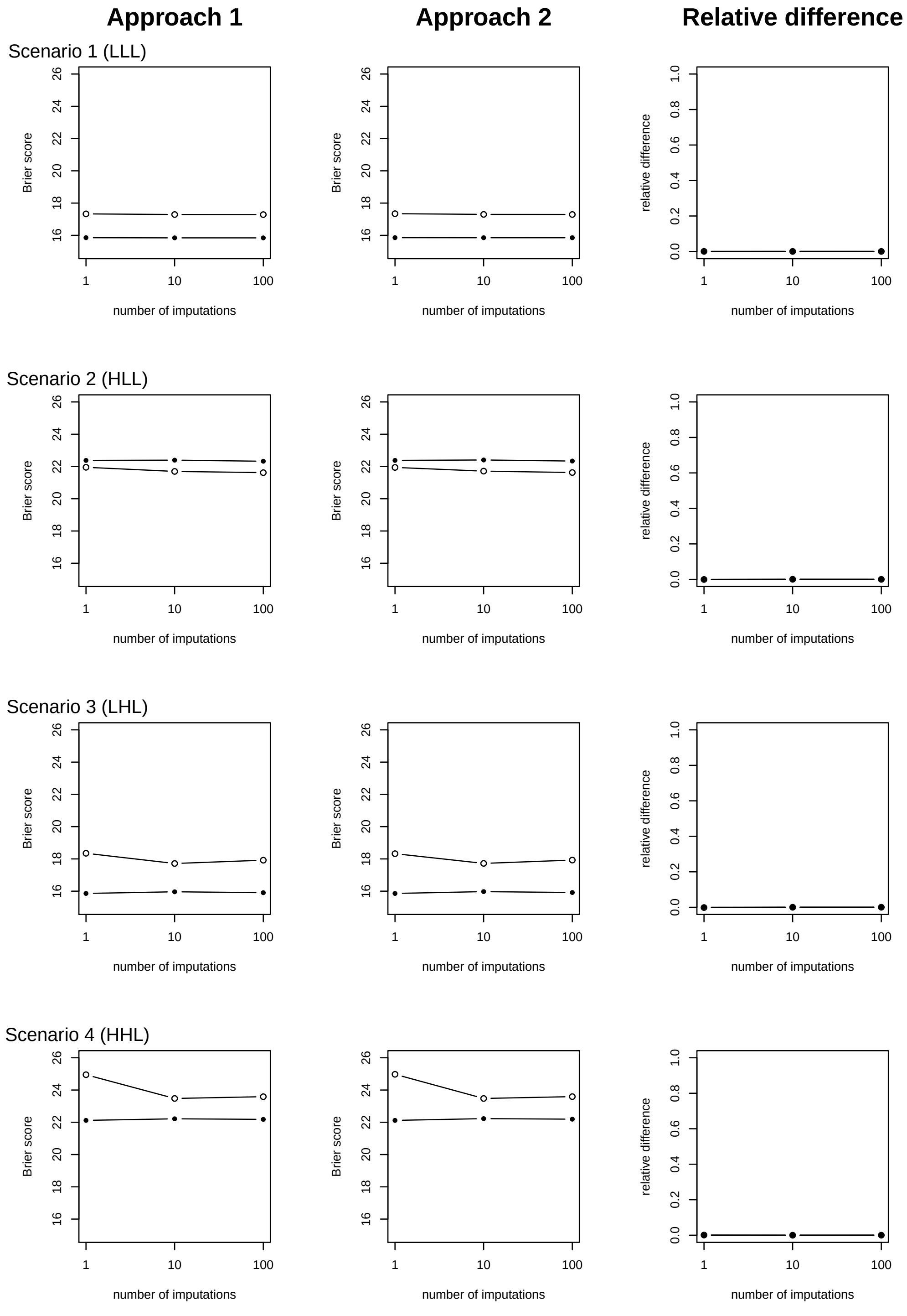

Figure S9: Average Brier scores ($\overline{\overline{B}}$). The four rows of plots from top to bottom correspond to simulation scenarios 1 to 4, MAR (tables S5 to S7). The two left columns of plots show results from approaches 1 and 2 versus the number of imputations used in the calibration of the predictors. The right-side column of plots displays the corresponding relative reductions for approach 1 relative to approach 2. See table S1 for description of the scenarios.

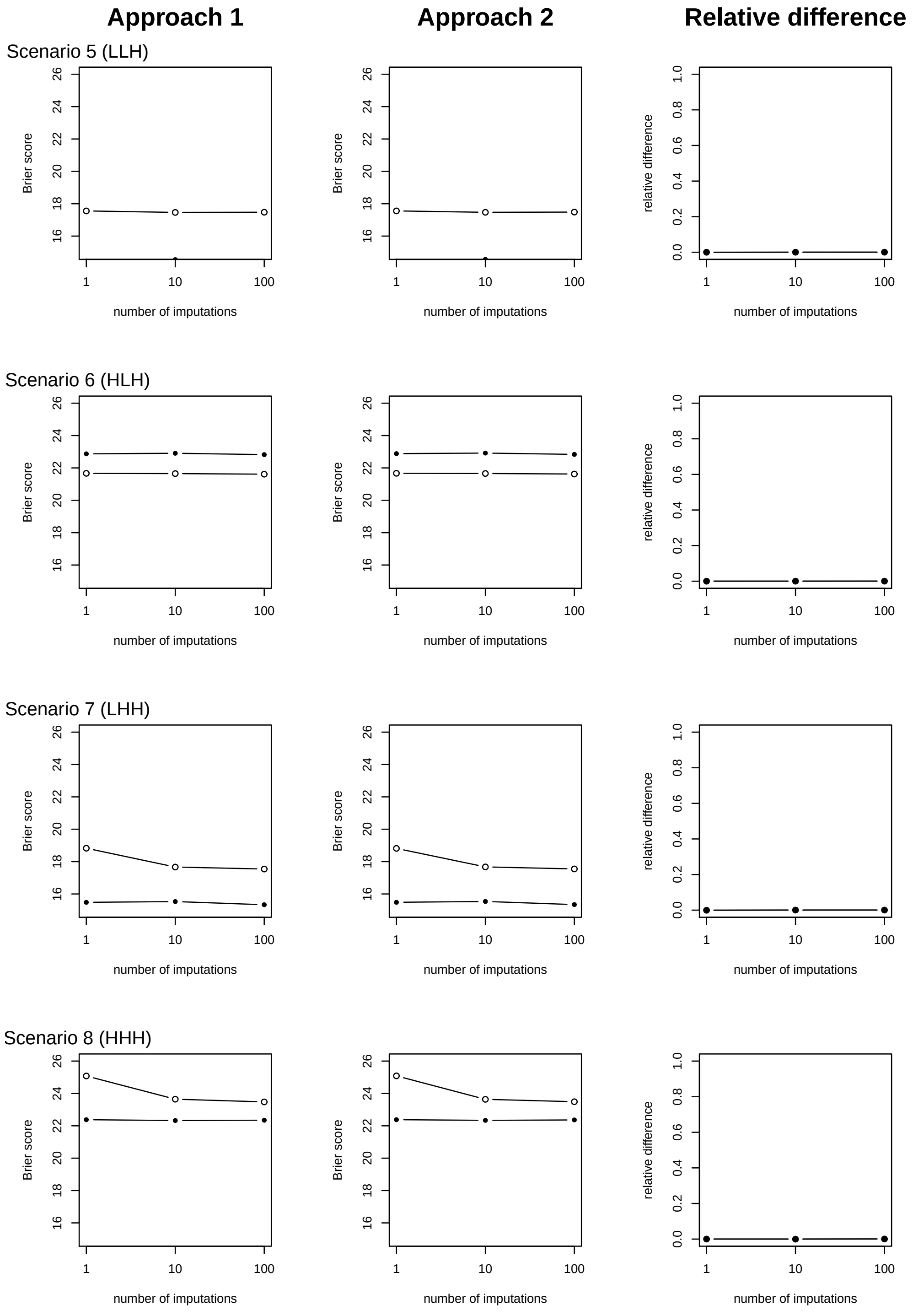

Figure S10: Average Brier scores ($\overline{\overline{B}}$). The four rows of plots from top to bottom correspond to simulation scenarios 5 to 8, MAR (tables S5 to S7). The two left columns of plots show results from approaches 1 and 2 versus the number of imputations used in the calibration of the predictors. The right-side column of plots displays the corresponding relative reductions for approach 1 relative to approach 2. See table S1 for description of the scenarios.

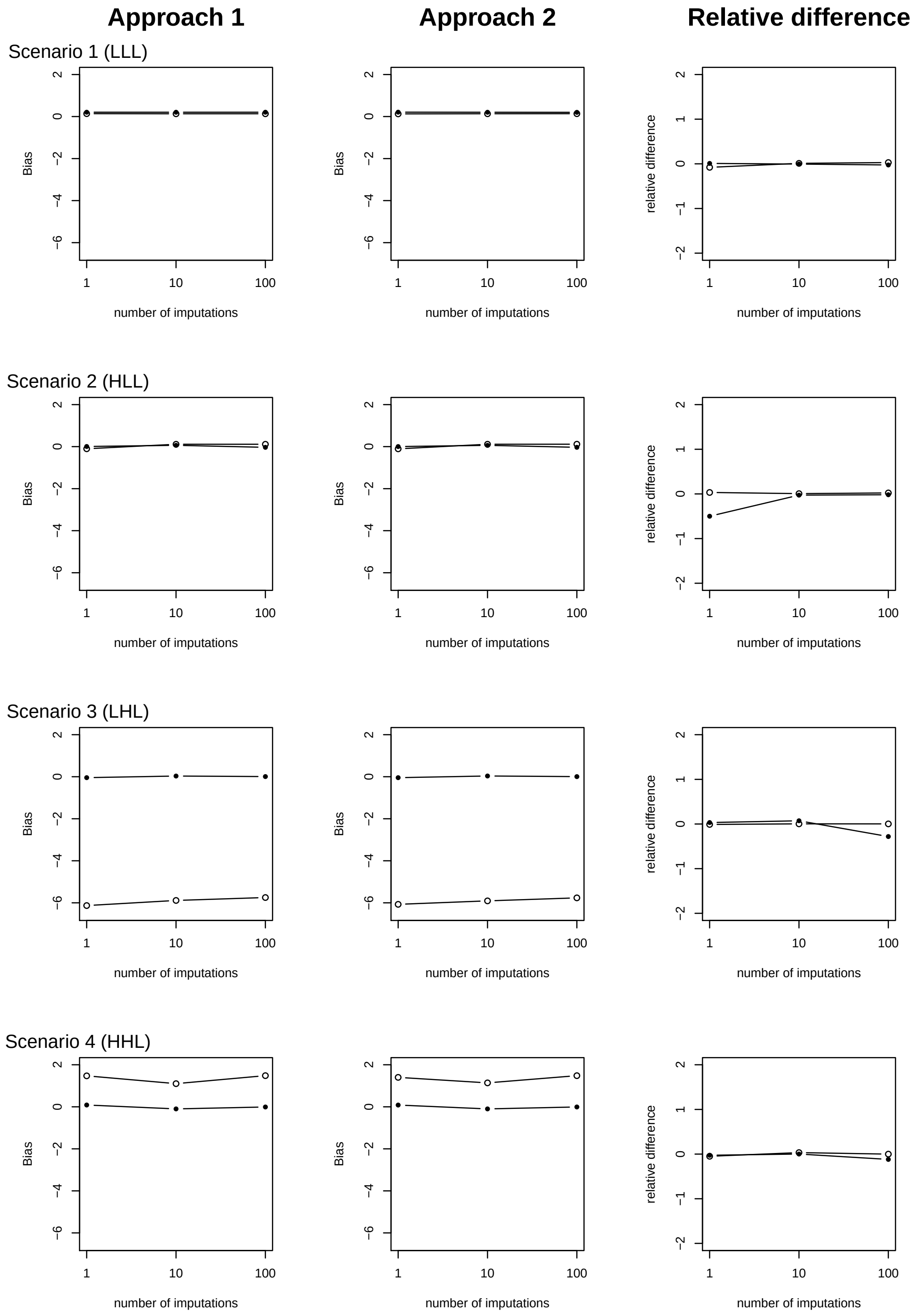

Figure S11: Average bias measures ($\overline{\overline{Bias}}$). The four rows of plots from top to bottom correspond to simulation scenarios 1 to 4, MAR (tables S5 to S7). The two left columns of plots show results from approaches 1 and 2 versus the number of imputations used in the calibration of the predictors. The right-side column of plots displays the corresponding relative reductions for approach 1 relative to approach 2. See table S1 for description of the scenarios.

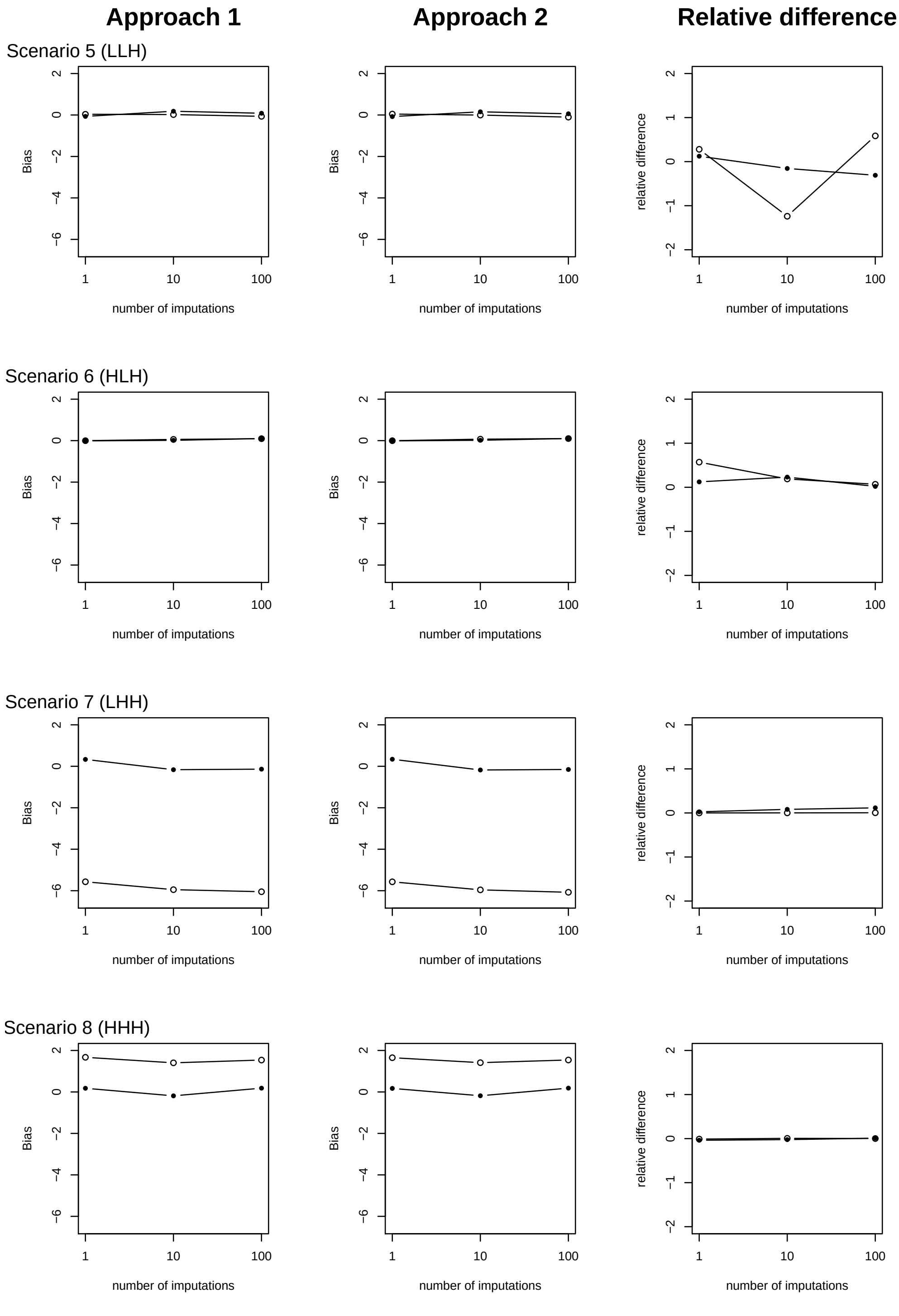

Figure S12: Average bias measures ($\overline{\overline{Bias}}$). The four rows of plots from top to bottom correspond to simulation scenarios 5 to 8, MAR (tables S5 to S7). The two left columns of plots show results from approaches 1 and 2 versus the number of imputations used in the calibration of the predictors. The right-side column of plots displays the corresponding relative reductions for approach 1 relative to approach 2. See table S1 for description of the scenarios.
