# Supplementary material for: Construction and assessment of prediction rules for binary outcome in the presence of missing predictor data using multiple imputation and cross‐validation: Methodological approach and data‐based evaluation
Source: Biom J. 2020 Feb 13;62(3):724–41. doi: 10.1002/bimj.201800289 (PMC7217034; doi:10.1002/bimj.201800289)
Supplement: Supplementary file 2 — SUPPORTING INFORMATION [file BIMJ-62-724-s001.zip › Code_and_Data/README.rtf]

Code description for the paper “Construction and assessment of prediction rules for binary outcome in the presence of missing predictor data using multiple imputation and cross-validation: methodological approach and data-based evaluation.”
Authors: Bart J. A. Mertens, Erika Banzato, Liesbeth C. de Wreede
All authors have contributed to code writing.
Email for questions or comments: b.mertens@lumc.nl
All code and data analysis was implemented and carried out with the statistical analysis package R.
R version 3.6.0 (2019-04-26)
Platform: x86_64-w64-mingw32/x64 (64-bit)
Running under: Windows 10 x64 (build 18362)

Matrix products: default

locale:
[1] LC_COLLATE=English_Netherlands.1252  LC_CTYPE=English_Netherlands.1252    LC_MONETARY=English_Netherlands.1252
[4] LC_NUMERIC=C                         LC_TIME=English_Netherlands.1252    

attached base packages:
[1] stats     graphics  grDevices utils     datasets  methods   base     

other attached packages:
[1] mice_3.5.0      lattice_0.20-38

loaded via a namespace (and not attached):
 [1] Rcpp_1.0.1        rstudioapi_0.10   magrittr_1.5      splines_3.6.0     MASS_7.3-51.4     tidyselect_0.2.5  R6_2.4.0         
 [8] rlang_0.4.0       jomo_2.6-8        minqa_1.2.4       dplyr_0.8.2       tools_3.6.0       parallel_3.6.0    nnet_7.3-12      
[15] grid_3.6.0        mitml_0.3-7       broom_0.5.2       nlme_3.1-140      pan_1.6           survival_2.44-1.1 lme4_1.1-21      
[22] assertthat_0.2.1  tibble_2.1.3      crayon_1.3.4      Matrix_1.2-17     nloptr_1.2.1      purrr_0.3.2       tidyr_0.8.3      
[29] rpart_4.1-15      glue_1.3.1        compiler_3.6.0    pillar_1.4.2      generics_0.0.2    backports_1.1.4   boot_1.3-22      
[36] pkgconfig_2.0.2 


Code content
The code submission details analysis of the CLL data and the presented simulation study. We first document the CLL data analysis and the simulation study next.
Length of computations
Note that the full set of codes presented here is highly computationally intensive and most likely requires days to run on a current desktop PC. The results produced by the steps describing the below analyses are saved to files as discussed in the below text and can be found in the subfolder(s) “Intermediate”. 
For the CLL data analysis, particularly step 1 is time-consuming (also because of the need to run replications of the analysis, especially for high numbers of imputations). If you want to avoid this, then put the intermediate files corresponding to the output from step 1 (files Binrep_...  .rps) into the working folder and proceed with steps 2 and 3 from there.  
CLL data analysis
Step 1. 
Note this step is highly computationally intensive.  The analysis begins with first generating 10 replicates of cross-validated predictions using methods 1, 2 and 3 (described in the paper – see section 3 for definition, as well as flow-charts in figures 1 and 2) and stores these results to disk. This analysis is carried out in file 
Approaches_1_2_3_forCLL_Means_Replicates.R
The file has the following structure.  At the top of the file, the original CLL data file (CLL_BMJ.rds) is read in and an administrative censoring is applied at 12 months. To prepare the data file for binary outcome analysis, the censoring variable is renamed to “Status” and the time variable is removed. The prepared data file is saved to disk (finaldata_CLL_bin.rds).
The next part of the file consists of two sections. In the first, replicate cross-validated predictions are generated for approach 1 first and results saved. In the second, cross-validation predictions are computed for approaches 2 and 3 with a separate piece of code and results again saved. At the start of each calculation the workspace is first cleared and the prepared data (finaldata_CLL_bin.rds) loaded. The number of folds for the cross-validation (constant “K”), as well as the number of replicates (value “REP”) of the analysis are always set to 10. The number of imputations must be manually changed to either 10, 100 or 1000 to obtain results shown in the paper. This is done by setting the constant “M” to the required value (indicated in the code) and then re-running the code. The multiple imputed predictions are averaged for each patient and for each replication and the results stored in the matrices
Binrep_1   	(for approach 1 – 694x10 dimensional matrix – each column contains the means of a replication)
Binrep_2	(for approach 2 – 694x10 dimensional matrix – each column contains the means of a replication)
Binrep_3	(for approach 3 – 694x10 dimensional matrix – each column contains the means of a replication)
These results are saved in the files 
Binrep_1_CLL_k10_m10.rds (for 10 imputations)
Binrep_1_CLL_k10_m100.rds (for 100 imputations)
Binrep_1_CLL_k10_m1000.rds (for 1000 imputations)
for approach 1,  and similarly for approaches 2 and 3:
Binrep_2_CLL_k10_m10.rds (for 10 imputations) – approach2
Binrep_2_CLL_k10_m100.rds (for 100 imputations) 
Binrep_2_CLL_k10_m1000.rds (for 1000 imputations)
Binrep_3_CLL_k10_m10.rds (for 10 imputations) – approach 3
Binrep_3_CLL_k10_m100.rds (for 100 imputations)
Binrep_3_CLL_k10_m1000.rds (for 1000 imputations)
In addition to the above results, we also save the complete set of predictions generated with approach 1 for M=1000. This result is stored in object “BINREPS” which is a 694x1000x10 dimensional array (storing predictions for observations (694) x imputation (1000) x replication (10)). The object is saved to disk in file 
BINREPS_1_CLL_k10_m1000.rds

Step 2. 
The next step in analysis is to load the generated predictions from step 1 and calculate summary measures (Brier scores and Variance measures (R statistic) – see section 5.1 for definition) from these. This is done in file
SummaryMeasuresScript_CLL_Variance.R
for the variance measures and file
SummaryMeasuresScript_CLL_Brier.R
for the Brier scores. 
The structure of file SummaryMeasuresScript_CLL_Variance.R  is as follows:
A matrix “resmat” is first initialized to store the calculated statistics and saved to disk. The first column contains the R measure, the second, third and fourth the 10%, 50% and 90% percentiles as discussed in the paper (section 5.1). Column 5 contains an indicator denoting whether the calculation is for a record containing missing values (1) or not (0). Column 6 contains an indicator for the prediction approach (1, 2 or 3). The final column contains the number of imputations on which the summary measure is based (10, 100 or 1000). 
In the remainder of the file the following calculation is repeated across all approaches and all numbers of imputations. First the workspace is cleared and the current version of the results matrix reloaded. One  of the 9 results files described end of step 1. is then loaded and the calculation of the R measure executed on the replicated predictions. The calculation is carried out separately on records with and without missing values and stored to the results matrix, together with the info in columns 5 to 7 on method, missing status and number of imputations. The matrix with summary variance measures is saved as 
resmat_CLL_Bin.rds
The structure of file SummaryMeasuresScript_CLL_Brier.R  is similar and as follows: 
A matrix “clldataresmat” is first initialized to store the calculated statistics and saved to disk. The first column contains the Brier statistic and the second the standard deviation of Brier scores. Column 3 contains an indicator denoting whether the calculation is for a record containing missing values (1) or not (0). Column 4 contains an indicator for the prediction approach (1, 2 or 3). The final column contains the number of imputations on which the summary measure is based (10, 100 or 1000).
In the remainder of the file the following calculation is repeated across all approaches and all numbers of imputations. First the workspace is cleared and the current version of the results matrix reloaded. One  of the 9 results files described end of step 1. is then loaded and the calculation of the Brier measure executed on the replicated predictions. The calculation is carried out separately on records with and without missing values and stored to the results matrix, together with the info in columns 3 to 5 on method, missing status and number of imputations. The matrix with summary Brier measures is saved as
clldataresmat_CLL_Bin_MEAN.rds
For the Brier score calculations, we also generate the statistics for M=1 (single imputation).  This calculation can be found at the end of the file SummaryMeasuresScript_CLL_Brier.R. 
The calculation again proceeds by first initializing a results matrix “clldataresmatsingle”.  Next,  the predictions from file BINREPS_1_CLL_k10_m1000.rds are loaded and only the first imputation record is retained from this data.  The calculation then proceeds along same lines as above and saved to file as 
clldataresmat_CLL_Bin_MEAN_single.rds

Step 3.
In this final step the calculated summary measures from step 2 are loaded in a clean session and used to compile the plots of figures 5 and 6 in the paper. See file 
MakePics_CLL.R
for the code.
The first part of the file first clears the workspace and loads the results files 
clldataresmat_CLL_Bin_MEAN.rds  (Brier scores for M=10, 100 and 1000)
clldataresmat_CLL_Bin_MEAN_single.rds (Brier scores for M=1)
The data is first combined to make the format suitable for the matplot() function.  The Brier scores are then plotted versus number of imputations to produce figure 5.
The second part of the code first clears the workspace and loads the results file 
resmat_CLL_Bin.rds (R measures, for M=10, 100, 1000).
The data is first reformatted to make the format suitable for the matplot() function.  The Brier scores are then plotted versus number of imputations to produce figure 6.

Simulations
The set-up of the simulation study is described in the supplementary material, S1.1.
The code is structured in such a way that 100 datasets can be generated and analyzed in parallel by making use of a computer cluster. The files can be used as follows:
'one_simulation_logreg.R' sets the parameters for generating and analyzing one dataset for each of the eight scenarios. To reduce runtime, the number of replications, sample size or number of imputations can be reduced. MCAR or MAR and the number of imputations have to be set manually. Data are generated and analyzed in the function 'sim_scenario'.
  'sim_scenario' is found in 'sim_scenario_MCAR_logreg.R' or 'sim_scenario_MAR_logreg.R'. After the generation of the dataset, data are set to missing according to the chosen mechanism. It is analyzed 10 times according to the four approaches (A1, A2, N1, N2). The output contains both the generated dataset and the cross-validated predictions under the different approaches for different replications.
  Tables S2-S7 have been created by summarizing over individuals, replications and simulations. The code for creating the summary statistics is given in 'SummaryMeasures_function_logreg_ext.R' and 'SummaryMeasures_FinalTable_logreg_MCAR_M10.R' (again manually setting MCAR or MAR and the number of imputations) (*). The tables are further formatted in MakeTablesinR.R.
Figures 7 and S1-6 have been generated by MakePicsinR_MCAR_vars.R, …Bias…, …Brier… (for MAR, replace MCAR by MAR in all files to create Figs S7-12). 
Intermediate results
For each of the missingness scenarios (MCAR or MAR) and each number of imputations (1, 10, 100), the  100 .Rdata-files containing the output of a single simulation are available on request from the authors. The summary files created in (*) are included.

> sessionInfo()
R version 3.6.1 (2019-07-05)
Platform: x86_64-w64-mingw32/x64 (64-bit)
Running under: Windows 10 x64 (build 17134)

Matrix products: default

locale:
[1] LC_COLLATE=Dutch_Netherlands.1252  LC_CTYPE=Dutch_Netherlands.1252    LC_MONETARY=Dutch_Netherlands.1252 LC_NUMERIC=C                      
[5] LC_TIME=Dutch_Netherlands.1252    

attached base packages:
[1] stats     graphics  grDevices utils     datasets  methods   base     

other attached packages:
[1] xtable_1.8-4    pROC_1.15.3     mice_3.6.0      lattice_0.20-38 MASS_7.3-51.4  

loaded via a namespace (and not attached):
 [1] Rcpp_1.0.2        plyr_1.8.4        pillar_1.4.2      compiler_3.6.1    nloptr_1.2.1      tools_3.6.1       boot_1.3-22      
 [8] zeallot_0.1.0     rpart_4.1-15      lme4_1.1-21       lifecycle_0.1.0   tibble_2.1.3      nlme_3.1-140      pkgconfig_2.0.3  
[15] rlang_0.4.0       Matrix_1.2-17     rstudioapi_0.10   parallel_3.6.1    dplyr_0.8.3       generics_0.0.2    vctrs_0.2.0      
[22] grid_3.6.1        nnet_7.3-12       tidyselect_0.2.5  glue_1.3.1        R6_2.4.0          survival_2.44-1.1 mitml_0.3-7      
[29] minqa_1.2.4       tidyr_1.0.0       purrr_0.3.2       magrittr_1.5      backports_1.1.5   splines_3.6.1     assertthat_0.2.1 
[36] broom_0.5.2       crayon_1.3.4      pan_1.6           jomo_2.6-9     
